# Supplementary material for: MEME-LaB: motif analysis in clusters
Source: Bioinformatics. 2013 May 14;29(13):1696–7. doi: 10.1093/bioinformatics/btt248 (PMC3694638; doi:10.1093/bioinformatics/btt248)
Supplement: Supplementary Data [file supp_btt248_Supplementary_Data.zip › Supplementary_Data/Results_Files/results.html]

MEME-LaB Results


MEME-LaB Results

This analysis was run with the following input parameters

|  |  |  |  |
| --- | --- | --- | --- |
| Gene list | 44botrytisclusters.txt | Sequences from | TAIR10\_upstream\_500\_20101028.fasta |
| Promoter maximum length | 500 | Number of motifs per cluster | 5 |
| Minimum motif width | 6 | Maximum motif width | 12 |

**Find**

**Filter motifs**

*You can filter the motifs displayed by the following criteria*

- Minimum number of promoters that the motif appears in
- Minimum percentage of promoters that the motif appears in
- Minimum information content more...
- Maximum p-value for positional bias more...
- Maximum p-value for strand bias more...
- Minimum distance score compared to a known PSSM

Promoters >=

% Promoters >=

Info content >=

Positional bias <=

Strand bias <=

Min distance >=

Clear

Apply

No motifs meet the specified criteria


Cluster 1 Motif 1

| Motif Featuresmore... | |
| --- | --- |
| Sites | 136 (80.9%) |
| Width | 12 |
| Information content | 13.6 |
| E-value | 8e-57 |
| Positional bias | 0.013 |
| Strand bias | 0.036 |

| Distance scores for most similar known PSSMs | |
| --- | --- |
| R05016 | 10.5 |
| R02187 | 10.8 |
| S-000067 | 10.9 |
| M01136 | 11.3 |
| M01126 | 11.5 |


Cluster 1 Motif 2

| Motif Featuresmore... | |
| --- | --- |
| Sites | 165 (98.2%) |
| Width | 12 |
| Information content | 11.5 |
| E-value | 1.3e-13 |
| Positional bias | 0.94 |
| Strand bias | 0.27 |

| Distance scores for most similar known PSSMs | |
| --- | --- |
| HK-00008 | 7.0 |
| EVE-0008 | 7.0 |
| M01216 | 8.0 |
| S-000477 | 8.0 |


Cluster 1 Motif 3

| Motif Featuresmore... | |
| --- | --- |
| Sites | 25 (14.8%) |
| Width | 8 |
| Information content | 12.5 |
| E-value | 1.6e-05 |
| Positional bias | 0.0023 |
| Strand bias | 0.35 |

| Distance scores for most similar known PSSMs | |
| --- | --- |
| S-000119 | 1.5 |
| S-000041 | 2.6 |
| S-000229 | 2.7 |
| S-000406 | 2.7 |
| M00366 | 2.9 |


Cluster 1 Motif 4

| Motif Featuresmore... | |
| --- | --- |
| Sites | 45 (26.7%) |
| Width | 11 |
| Information content | 12.9 |
| E-value | 0.016 |
| Positional bias | 0.11 |
| Strand bias | 0.068 |

| Distance scores for most similar known PSSMs | |
| --- | --- |
| M00658 | 9.7 |
| S-000159 | 10.2 |
| M01295 | 10.4 |
| M01172 | 10.7 |
| M01126 | 11.4 |


Cluster 1 Motif 5

| Motif Featuresmore... | |
| --- | --- |
| Sites | 43 (25.5%) |
| Width | 8 |
| Information content | 11.6 |
| E-value | 4.6e-06 |
| Positional bias | 1.2e-05 |
| Strand bias | 0.5 |

| Distance scores for most similar known PSSMs | |
| --- | --- |
| S-000474 | 2.6 |
| S-000471 | 5.6 |
| S-000483 | 5.7 |
| M00967 | 7.4 |
| S-000115 | 9.8 |

  


Cluster 2 Motif 1

| Motif Featuresmore... | |
| --- | --- |
| Sites | 42 (100%) |
| Width | 11 |
| Information content | 12.4 |
| E-value | 0.0002 |
| Positional bias | 0.79 |
| Strand bias | 0.022 |

| Distance scores for most similar known PSSMs | |
| --- | --- |
| M01216 | 7.0 |
| S-000477 | 7.1 |
| HK-00008 | 8.5 |
| EVE-0008 | 8.5 |


Cluster 2 Motif 2

| Motif Featuresmore... | |
| --- | --- |
| Sites | 38 (90.4%) |
| Width | 12 |
| Information content | 12.5 |
| E-value | 0.0072 |
| Positional bias | 0.42 |
| Strand bias | 0.44 |

| Distance scores for most similar known PSSMs | |
| --- | --- |
| R05016 | 9.9 |
| MA-00391 | 10.5 |
| M01126 | 10.5 |
| M00632 | 10.6 |
| M01136 | 10.6 |


Cluster 2 Motif 3

| Motif Featuresmore... | |
| --- | --- |
| Sites | 10 (23.8%) |
| Width | 12 |
| Information content | 15.3 |
| E-value | 0.52 |
| Positional bias | 0.027 |
| Strand bias | 0.055 |

| Distance scores for most similar known PSSMs | |
| --- | --- |
| M00368 | 4.2 |
| M00366 | 5.0 |
| M00942 | 5.0 |
| M00367 | 5.2 |
| M00441 | 5.5 |


Cluster 2 Motif 4

| Motif Featuresmore... | |
| --- | --- |
| Sites | 26 (61.9%) |
| Width | 6 |
| Information content | 9.2 |
| E-value | 1.4e+02 |
| Positional bias | 0.36 |
| Strand bias | 0.084 |

| Distance scores for most similar known PSSMs | |
| --- | --- |
| S-000474 | 2.3 |
| S-000483 | 2.4 |
| S-000471 | 2.6 |
| M00967 | 6.0 |
| S-000216 | 7.1 |


Cluster 2 Motif 5

| Motif Featuresmore... | |
| --- | --- |
| Sites | 13 (30.9%) |
| Width | 11 |
| Information content | 13.0 |
| E-value | 4.5e+03 |
| Positional bias | 0.2 |
| Strand bias | 0.13 |

| Distance scores for most similar known PSSMs | |
| --- | --- |
| S-000492 | 10.1 |
| S-000167 | 11.5 |
| M01113 | 12.0 |
| S-000479 | 12.0 |
| S-000138 | 12.1 |

  


Cluster 3 Motif 1

| Motif Featuresmore... | |
| --- | --- |
| Sites | 67 (100%) |
| Width | 12 |
| Information content | 14.2 |
| E-value | 5.7e-21 |
| Positional bias | 0.56 |
| Strand bias | 0.071 |

| Distance scores for most similar known PSSMs | |
| --- | --- |
| S-000067 | 9.3 |
| HK-00008 | 9.6 |
| EVE-0008 | 9.6 |
| M01084 | 10.0 |


Cluster 3 Motif 2

| Motif Featuresmore... | |
| --- | --- |
| Sites | 24 (35.8%) |
| Width | 11 |
| Information content | 13.2 |
| E-value | 0.071 |
| Positional bias | 0.036 |
| Strand bias | 0.58 |

| Distance scores for most similar known PSSMs | |
| --- | --- |
| S-000460 | 5.4 |
| MA-02051 | 9.4 |
| S-000486 | 10.2 |
| M01295 | 10.6 |
| S-000395 | 10.8 |


Cluster 3 Motif 3

| Motif Featuresmore... | |
| --- | --- |
| Sites | 8 (11.9%) |
| Width | 11 |
| Information content | 14.0 |
| E-value | 3.1e+03 |
| Positional bias | 0.63 |
| Strand bias | 0.36 |

| Distance scores for most similar known PSSMs | |
| --- | --- |
| RJH-00005 | 12.1 |
| HK-00033 | 14.9 |
| MA-03581 | 15.0 |
| R02251 | 15.2 |
| S-000292 | 15.5 |


Cluster 3 Motif 4

| Motif Featuresmore... | |
| --- | --- |
| Sites | 8 (11.9%) |
| Width | 11 |
| Information content | 14.4 |
| E-value | 2.7e+04 |
| Positional bias | 0.81 |
| Strand bias | 0.14 |

| Distance scores for most similar known PSSMs | |
| --- | --- |
| M00479 | 11.9 |
| M00732 | 13.3 |
| MA-00792 | 13.7 |
| M00308 | 13.8 |
| M01219 | 13.8 |


Cluster 3 Motif 5

| Motif Featuresmore... | |
| --- | --- |
| Sites | 20 (29.8%) |
| Width | 11 |
| Information content | 13.3 |
| E-value | 6.4e+02 |
| Positional bias | 0.06 |
| Strand bias | 0.41 |

| Distance scores for most similar known PSSMs | |
| --- | --- |
| S-000462 | 9.3 |
| S-000468 | 9.3 |
| M00632 | 10.2 |
| S-000270 | 11.7 |
| S-000424 | 11.9 |

  


Cluster 4 Motif 1

| Motif Featuresmore... | |
| --- | --- |
| Sites | 16 (72.7%) |
| Width | 11 |
| Information content | 13.8 |
| E-value | 7e-11 |
| Positional bias | 0.0068 |
| Strand bias | 0.6 |

| Distance scores for most similar known PSSMs | |
| --- | --- |
| M00441 | 3.7 |
| M00368 | 4.0 |
| M00942 | 4.4 |
| M00440 | 4.9 |
| M00366 | 4.9 |


Cluster 4 Motif 2

| Motif Featuresmore... | |
| --- | --- |
| Sites | 22 (100%) |
| Width | 11 |
| Information content | 11.4 |
| E-value | 3.9 |
| Positional bias | 0.9 |
| Strand bias | 0.42 |

| Distance scores for most similar known PSSMs | |
| --- | --- |
| M00456 | 9.8 |
| M00257 | 10.0 |
| S-000148 | 10.7 |
| MA-01331 | 10.7 |
| M01082 | 10.8 |


Cluster 4 Motif 3

| Motif Featuresmore... | |
| --- | --- |
| Sites | 22 (100%) |
| Width | 12 |
| Information content | 13.5 |
| E-value | 2.9 |
| Positional bias | 0.49 |
| Strand bias | 0.067 |

| Distance scores for most similar known PSSMs | |
| --- | --- |
| S-000067 | 10.4 |
| MA-00491 | 11.2 |
| M00022 | 11.2 |
| HK-00008 | 11.9 |
| EVE-0008 | 11.9 |


Cluster 4 Motif 4

| Motif Featuresmore... | |
| --- | --- |
| Sites | 2 (9%) |
| Width | 11 |
| Information content | 19.4 |
| E-value | 4.8e+04 |
| Positional bias | 0.032 |
| Strand bias | 0.25 |

| Distance scores for most similar known PSSMs | |
| --- | --- |
| R02123 | 12.0 |
| MA-00481 | 15.1 |
| M00175 | 15.2 |
| MA-04271 | 15.4 |
| M00005 | 15.5 |


Cluster 4 Motif 5

| Motif Featuresmore... | |
| --- | --- |
| Sites | 2 (9%) |
| Width | 7 |
| Information content | 12.4 |
| E-value | 6.4e+04 |
| Positional bias | 0.029 |
| Strand bias | 0.75 |

| Distance scores for most similar known PSSMs | |
| --- | --- |
| MA-03611 | 7.1 |
| M01546 | 7.9 |
| M01096 | 8.1 |
| S-000430 | 8.2 |
| MA-04271 | 8.6 |

  


Cluster 5 Motif 1

| Motif Featuresmore... | |
| --- | --- |
| Sites | 74 (100%) |
| Width | 12 |
| Information content | 14.5 |
| E-value | 3.5e-21 |
| Positional bias | 0.29 |
| Strand bias | 0.04 |

| Distance scores for most similar known PSSMs | |
| --- | --- |
| S-000067 | 6.8 |
| S-000477 | 8.8 |
| HK-00008 | 9.0 |
| EVE-0008 | 9.0 |


Cluster 5 Motif 2

| Motif Featuresmore... | |
| --- | --- |
| Sites | 27 (36.4%) |
| Width | 8 |
| Information content | 11.1 |
| E-value | 5e-05 |
| Positional bias | 0.016 |
| Strand bias | 0.5 |

| Distance scores for most similar known PSSMs | |
| --- | --- |
| M00366 | 1.5 |
| S-000119 | 2.1 |
| S-000394 | 2.1 |
| MA-03101 | 2.2 |
| M00368 | 2.4 |


Cluster 5 Motif 3

| Motif Featuresmore... | |
| --- | --- |
| Sites | 39 (52.7%) |
| Width | 12 |
| Information content | 12.8 |
| E-value | 0.01 |
| Positional bias | 0.05 |
| Strand bias | 0.5 |

| Distance scores for most similar known PSSMs | |
| --- | --- |
| M01295 | 8.7 |
| MA-02051 | 9.7 |
| M01021 | 10.5 |
| S-000462 | 10.5 |
| S-000468 | 10.5 |


Cluster 5 Motif 4

| Motif Featuresmore... | |
| --- | --- |
| Sites | 7 (9.4%) |
| Width | 12 |
| Information content | 16.0 |
| E-value | 2.7e+03 |
| Positional bias | 0.62 |
| Strand bias | 0.062 |

| Distance scores for most similar known PSSMs | |
| --- | --- |
| R02207 | 15.3 |
| M00327 | 15.4 |
| M00378 | 15.7 |
| S-000462 | 15.7 |
| S-000468 | 15.7 |


Cluster 5 Motif 5

| Motif Featuresmore... | |
| --- | --- |
| Sites | 38 (51.3%) |
| Width | 8 |
| Information content | 13.7 |
| E-value | 9.2e+03 |
| Positional bias | 0.006 |
| Strand bias | 0.44 |

| Distance scores for most similar known PSSMs | |
| --- | --- |
| S-000385 | 1.8 |
| M00142 | 7.0 |
| S-000098 | 7.1 |
| S-000039 | 7.4 |
| S-000149 | 7.6 |

  


Cluster 6 Motif 1

| Motif Featuresmore... | |
| --- | --- |
| Sites | 96 (95%) |
| Width | 12 |
| Information content | 13.8 |
| E-value | 1.5e-27 |
| Positional bias | 0.38 |
| Strand bias | 0.24 |

| Distance scores for most similar known PSSMs | |
| --- | --- |
| HK-00008 | 8.3 |
| EVE-0008 | 8.3 |
| S-000067 | 9.5 |
| M00456 | 10.1 |


Cluster 6 Motif 2

| Motif Featuresmore... | |
| --- | --- |
| Sites | 30 (29.7%) |
| Width | 12 |
| Information content | 12.8 |
| E-value | 0.012 |
| Positional bias | 0.075 |
| Strand bias | 0.18 |

| Distance scores for most similar known PSSMs | |
| --- | --- |
| S-000460 | 7.1 |
| M00723 | 9.6 |
| M01295 | 10.9 |
| MA-02051 | 11.0 |
| M00658 | 11.2 |


Cluster 6 Motif 3

| Motif Featuresmore... | |
| --- | --- |
| Sites | 25 (24.7%) |
| Width | 12 |
| Information content | 11.8 |
| E-value | 3 |
| Positional bias | 0.00016 |
| Strand bias | 0.5 |

| Distance scores for most similar known PSSMs | |
| --- | --- |
| M00366 | 4.7 |
| MA-01281 | 5.1 |
| M01584 | 5.3 |
| M01034 | 5.3 |
| M00367 | 5.6 |


Cluster 6 Motif 4

| Motif Featuresmore... | |
| --- | --- |
| Sites | 35 (34.6%) |
| Width | 12 |
| Information content | 13.1 |
| E-value | 8e+03 |
| Positional bias | 0.68 |
| Strand bias | 0.0083 |

| Distance scores for most similar known PSSMs | |
| --- | --- |
| M00769 | 9.4 |
| M00456 | 9.5 |
| JC-000001 | 10.0 |
| M00722 | 10.4 |
| S-000408 | 10.4 |


Cluster 6 Motif 5

| Motif Featuresmore... | |
| --- | --- |
| Sites | 3 (2.9%) |
| Width | 11 |
| Information content | 16.9 |
| E-value | 1.8e+05 |
| Positional bias | 0.32 |
| Strand bias | 0.12 |

| Distance scores for most similar known PSSMs | |
| --- | --- |
| M00948 | 12.2 |
| S-000217 | 12.5 |
| MA-04041 | 13.1 |
| S-000360 | 15.0 |
| S-000351 | 15.0 |

  


  


Cluster 8 Motif 1

| Motif Featuresmore... | |
| --- | --- |
| Sites | 103 (100%) |
| Width | 12 |
| Information content | 13.8 |
| E-value | 3.8e-29 |
| Positional bias | 0.61 |
| Strand bias | 0.0028 |

| Distance scores for most similar known PSSMs | |
| --- | --- |
| S-000067 | 8.1 |
| HK-00008 | 8.7 |
| EVE-0008 | 8.7 |
| S-000477 | 8.7 |


Cluster 8 Motif 2

| Motif Featuresmore... | |
| --- | --- |
| Sites | 23 (22.3%) |
| Width | 10 |
| Information content | 12.1 |
| E-value | 0.0039 |
| Positional bias | 0.00012 |
| Strand bias | 0.0053 |

| Distance scores for most similar known PSSMs | |
| --- | --- |
| M00368 | 3.1 |
| S-000505 | 3.2 |
| M00366 | 3.4 |
| S-000041 | 3.4 |
| S-000119 | 3.6 |


Cluster 8 Motif 3

| Motif Featuresmore... | |
| --- | --- |
| Sites | 19 (18.4%) |
| Width | 8 |
| Information content | 12.7 |
| E-value | 5.1 |
| Positional bias | 0.22 |
| Strand bias | 0.5 |

| Distance scores for most similar known PSSMs | |
| --- | --- |
| S-000471 | 4.5 |
| S-000474 | 6.3 |
| S-000483 | 6.4 |
| M01123 | 8.6 |
| M01013 | 9.3 |


Cluster 8 Motif 4

| Motif Featuresmore... | |
| --- | --- |
| Sites | 2 (1.9%) |
| Width | 10 |
| Information content | 16.8 |
| E-value | 2.4e+05 |
| Positional bias | 0.76 |
| Strand bias | 0.75 |

| Distance scores for most similar known PSSMs | |
| --- | --- |
| M00644 | 12.6 |
| MA-00481 | 13.2 |
| R02123 | 13.7 |
| M00176 | 13.9 |
| M00361 | 14.5 |


Cluster 8 Motif 5

| Motif Featuresmore... | |
| --- | --- |
| Sites | 4 (3.8%) |
| Width | 11 |
| Information content | 16.3 |
| E-value | 4.5e+05 |
| Positional bias | 0.4 |
| Strand bias | 0.69 |

| Distance scores for most similar known PSSMs | |
| --- | --- |
| M01492 | 13.7 |
| S-000224 | 15.2 |
| S-000153 | 16.2 |
| M01282 | 16.6 |
| M00963 | 16.9 |

  


Cluster 9 Motif 1

| Motif Featuresmore... | |
| --- | --- |
| Sites | 292 (100%) |
| Width | 12 |
| Information content | 12.4 |
| E-value | 2e-108 |
| Positional bias | 0.0023 |
| Strand bias | 0.38 |

| Distance scores for most similar known PSSMs | |
| --- | --- |
| S-000067 | 9.1 |
| M01136 | 9.5 |
| M01021 | 9.6 |
| M01126 | 9.7 |
| S-000453 | 10.0 |


Cluster 9 Motif 2

| Motif Featuresmore... | |
| --- | --- |
| Sites | 92 (31.5%) |
| Width | 10 |
| Information content | 12.7 |
| E-value | 2.9e-43 |
| Positional bias | 8.3e-13 |
| Strand bias | 0.3 |

| Distance scores for most similar known PSSMs | |
| --- | --- |
| S-000471 | 2.2 |
| S-000474 | 2.5 |
| S-000483 | 5.1 |
| M00967 | 8.8 |
| M01238 | 8.9 |


Cluster 9 Motif 3

| Motif Featuresmore... | |
| --- | --- |
| Sites | 63 (21.5%) |
| Width | 12 |
| Information content | 13.6 |
| E-value | 2.3e-23 |
| Positional bias | 0.26 |
| Strand bias | 0.038 |

| Distance scores for most similar known PSSMs | |
| --- | --- |
| S-000460 | 6.5 |
| MA-02051 | 8.9 |
| S-000486 | 10.6 |
| M00723 | 10.7 |
| S-000462 | 10.8 |


Cluster 9 Motif 4

| Motif Featuresmore... | |
| --- | --- |
| Sites | 22 (7.5%) |
| Width | 12 |
| Information content | 15.5 |
| E-value | 3.8e-07 |
| Positional bias | 0.0001 |
| Strand bias | 0.14 |

| Distance scores for most similar known PSSMs | |
| --- | --- |
| RJH-00017 | 11.0 |
| MA-03491 | 11.6 |
| M00951 | 11.8 |
| S-000167 | 12.4 |
| MA-04041 | 12.8 |


Cluster 9 Motif 5

| Motif Featuresmore... | |
| --- | --- |
| Sites | 55 (18.8%) |
| Width | 10 |
| Information content | 12.9 |
| E-value | 2.9e-10 |
| Positional bias | 0.04 |
| Strand bias | 0.089 |

| Distance scores for most similar known PSSMs | |
| --- | --- |
| M00658 | 8.3 |
| S-000159 | 9.7 |
| M01172 | 10.1 |
| M00746 | 10.9 |
| M00971 | 11.1 |

  


Cluster 10 Motif 1

| Motif Featuresmore... | |
| --- | --- |
| Sites | 161 (87%) |
| Width | 12 |
| Information content | 13.1 |
| E-value | 1.4e-46 |
| Positional bias | 0.15 |
| Strand bias | 0.041 |

| Distance scores for most similar known PSSMs | |
| --- | --- |
| S-000477 | 8.2 |
| M01021 | 9.1 |
| M01136 | 9.4 |
| S-000067 | 9.5 |
| MA-00491 | 9.7 |


Cluster 10 Motif 2

| Motif Featuresmore... | |
| --- | --- |
| Sites | 53 (28.6%) |
| Width | 11 |
| Information content | 12.5 |
| E-value | 5.2e-07 |
| Positional bias | 9.4e-06 |
| Strand bias | 0.5 |

| Distance scores for most similar known PSSMs | |
| --- | --- |
| S-000471 | 3.4 |
| S-000474 | 3.5 |
| S-000483 | 6.3 |
| M01238 | 9.0 |
| S-000030 | 9.8 |


Cluster 10 Motif 3

| Motif Featuresmore... | |
| --- | --- |
| Sites | 22 (11.8%) |
| Width | 10 |
| Information content | 13.4 |
| E-value | 0.00023 |
| Positional bias | 3.1e-05 |
| Strand bias | 0.067 |

| Distance scores for most similar known PSSMs | |
| --- | --- |
| S-000041 | 2.1 |
| S-000119 | 2.8 |
| M00367 | 2.8 |
| M00369 | 3.6 |
| M00368 | 3.9 |


Cluster 10 Motif 4

| Motif Featuresmore... | |
| --- | --- |
| Sites | 15 (8.1%) |
| Width | 12 |
| Information content | 15.0 |
| E-value | 19 |
| Positional bias | 0.32 |
| Strand bias | 0.5 |

| Distance scores for most similar known PSSMs | |
| --- | --- |
| MA-02761 | 11.2 |
| MA-04041 | 12.4 |
| M00319 | 12.6 |
| MA-03491 | 12.7 |
| M01238 | 14.7 |


Cluster 10 Motif 5

| Motif Featuresmore... | |
| --- | --- |
| Sites | 36 (19.4%) |
| Width | 11 |
| Information content | 13.2 |
| E-value | 49 |
| Positional bias | 0.043 |
| Strand bias | 0.2 |

| Distance scores for most similar known PSSMs | |
| --- | --- |
| S-000473 | 9.5 |
| S-000159 | 10.1 |
| M00658 | 10.7 |
| M01126 | 11.7 |
| M00028 | 12.1 |

  


Cluster 11 Motif 1

| Motif Featuresmore... | |
| --- | --- |
| Sites | 324 (89%) |
| Width | 12 |
| Information content | 12.7 |
| E-value | 4.8e-131 |
| Positional bias | 0.028 |
| Strand bias | 0.082 |

| Distance scores for most similar known PSSMs | |
| --- | --- |
| MA-00451 | 9.3 |
| M01136 | 9.6 |
| R05016 | 10.1 |
| M01126 | 10.4 |
| S-000245 | 10.5 |


Cluster 11 Motif 2

| Motif Featuresmore... | |
| --- | --- |
| Sites | 113 (31%) |
| Width | 11 |
| Information content | 12.5 |
| E-value | 3.7e-47 |
| Positional bias | 1.7e-18 |
| Strand bias | 0.13 |

| Distance scores for most similar known PSSMs | |
| --- | --- |
| S-000471 | 1.6 |
| S-000474 | 2.3 |
| S-000483 | 4.9 |
| M01238 | 8.7 |
| M00967 | 8.9 |


Cluster 11 Motif 3

| Motif Featuresmore... | |
| --- | --- |
| Sites | 214 (58.7%) |
| Width | 12 |
| Information content | 13.1 |
| E-value | 6.8e-19 |
| Positional bias | 0.2 |
| Strand bias | 0.0025 |

| Distance scores for most similar known PSSMs | |
| --- | --- |
| HK-00008 | 8.9 |
| EVE-0008 | 8.9 |
| S-000067 | 9.6 |
| M00456 | 9.7 |


Cluster 11 Motif 4

| Motif Featuresmore... | |
| --- | --- |
| Sites | 48 (13.1%) |
| Width | 11 |
| Information content | 11.3 |
| E-value | 1.1e-05 |
| Positional bias | 0.068 |
| Strand bias | 0.56 |

| Distance scores for most similar known PSSMs | |
| --- | --- |
| M01057 | 9.3 |
| S-000146 | 10.1 |
| S-000205 | 10.1 |
| M01090 | 10.2 |
| MA-00792 | 10.5 |


Cluster 11 Motif 5

| Motif Featuresmore... | |
| --- | --- |
| Sites | 64 (17.5%) |
| Width | 11 |
| Information content | 13.1 |
| E-value | 5.8e-16 |
| Positional bias | 0.04 |
| Strand bias | 0.35 |

| Distance scores for most similar known PSSMs | |
| --- | --- |
| S-000362 | 9.5 |
| M00110 | 10.4 |
| RJH-00017 | 10.7 |
| M01238 | 10.8 |
| S-000167 | 10.8 |

  


Cluster 12 Motif 1

| Motif Featuresmore... | |
| --- | --- |
| Sites | 294 (73.3%) |
| Width | 12 |
| Information content | 13.5 |
| E-value | 2.6e-152 |
| Positional bias | 0.0025 |
| Strand bias | 0.027 |

| Distance scores for most similar known PSSMs | |
| --- | --- |
| R05016 | 9.5 |
| R02187 | 9.9 |
| MA-00391 | 10.3 |
| M01126 | 11.4 |
| M01136 | 11.5 |


Cluster 12 Motif 2

| Motif Featuresmore... | |
| --- | --- |
| Sites | 258 (64.3%) |
| Width | 12 |
| Information content | 13.7 |
| E-value | 3.7e-41 |
| Positional bias | 0.47 |
| Strand bias | 0.046 |

| Distance scores for most similar known PSSMs | |
| --- | --- |
| HK-00008 | 7.2 |
| EVE-0008 | 7.2 |
| S-000477 | 8.0 |
| R02164 | 8.5 |


Cluster 12 Motif 3

| Motif Featuresmore... | |
| --- | --- |
| Sites | 74 (18.4%) |
| Width | 11 |
| Information content | 12.9 |
| E-value | 1.2e-19 |
| Positional bias | 0.0022 |
| Strand bias | 0.36 |

| Distance scores for most similar known PSSMs | |
| --- | --- |
| S-000460 | 5.4 |
| S-000486 | 9.5 |
| MA-02051 | 9.5 |
| S-000499 | 10.5 |
| M00077 | 11.5 |


Cluster 12 Motif 4

| Motif Featuresmore... | |
| --- | --- |
| Sites | 38 (9.4%) |
| Width | 11 |
| Information content | 13.8 |
| E-value | 5e-14 |
| Positional bias | 3.2e-08 |
| Strand bias | 0.31 |

| Distance scores for most similar known PSSMs | |
| --- | --- |
| S-000041 | 2.5 |
| M00368 | 3.3 |
| S-000119 | 3.5 |
| M00367 | 3.8 |
| M00942 | 3.9 |


Cluster 12 Motif 5

| Motif Featuresmore... | |
| --- | --- |
| Sites | 59 (14.7%) |
| Width | 11 |
| Information content | 12.2 |
| E-value | 0.00049 |
| Positional bias | 0.036 |
| Strand bias | 0.5 |

| Distance scores for most similar known PSSMs | |
| --- | --- |
| RJH-00017 | 9.2 |
| MA-03491 | 10.4 |
| S-000167 | 10.8 |
| MA-02181 | 11.1 |
| M00951 | 11.1 |

  


Cluster 13 Motif 1

| Motif Featuresmore... | |
| --- | --- |
| Sites | 439 (81.2%) |
| Width | 12 |
| Information content | 13.9 |
| E-value | 5.8e-219 |
| Positional bias | 0.0024 |
| Strand bias | 0.043 |

| Distance scores for most similar known PSSMs | |
| --- | --- |
| S-000067 | 9.3 |
| MA-00491 | 10.2 |
| M00022 | 10.2 |
| HK-00008 | 10.2 |
| EVE-0008 | 10.2 |


Cluster 13 Motif 2

| Motif Featuresmore... | |
| --- | --- |
| Sites | 181 (33.5%) |
| Width | 8 |
| Information content | 11.3 |
| E-value | 1.2e-77 |
| Positional bias | 2.3e-17 |
| Strand bias | 0.23 |

| Distance scores for most similar known PSSMs | |
| --- | --- |
| S-000474 | 1.5 |
| S-000483 | 3.7 |
| M01238 | 7.9 |
| M00967 | 8.1 |
| MA-03811 | 8.4 |


Cluster 13 Motif 3

| Motif Featuresmore... | |
| --- | --- |
| Sites | 138 (25.5%) |
| Width | 12 |
| Information content | 13.5 |
| E-value | 7.2e-57 |
| Positional bias | 0.0071 |
| Strand bias | 0.1 |

| Distance scores for most similar known PSSMs | |
| --- | --- |
| S-000460 | 6.8 |
| R02187 | 9.5 |
| MA-02051 | 9.5 |
| M00723 | 10.1 |
| R05016 | 10.8 |


Cluster 13 Motif 4

| Motif Featuresmore... | |
| --- | --- |
| Sites | 100 (18.5%) |
| Width | 11 |
| Information content | 13.4 |
| E-value | 8.6e-32 |
| Positional bias | 0.0018 |
| Strand bias | 0.54 |

| Distance scores for most similar known PSSMs | |
| --- | --- |
| S-000159 | 7.2 |
| M00658 | 11.4 |
| M00046 | 13.0 |
| M00028 | 13.5 |
| M01126 | 13.7 |


Cluster 13 Motif 5

| Motif Featuresmore... | |
| --- | --- |
| Sites | 74 (13.7%) |
| Width | 12 |
| Information content | 14.1 |
| E-value | 1.1e-22 |
| Positional bias | 0.00029 |
| Strand bias | 0.15 |

| Distance scores for most similar known PSSMs | |
| --- | --- |
| RJH-00017 | 9.8 |
| S-000167 | 10.1 |
| S-000362 | 10.4 |
| M00486 | 11.7 |
| M00415 | 11.9 |

  


Cluster 14 Motif 1

| Motif Featuresmore... | |
| --- | --- |
| Sites | 276 (100%) |
| Width | 12 |
| Information content | 12.8 |
| E-value | 2.1e-94 |
| Positional bias | 0.17 |
| Strand bias | 0.066 |

| Distance scores for most similar known PSSMs | |
| --- | --- |
| S-000067 | 8.9 |
| HK-00008 | 9.3 |
| EVE-0008 | 9.3 |
| S-000477 | 9.3 |


Cluster 14 Motif 2

| Motif Featuresmore... | |
| --- | --- |
| Sites | 114 (41.3%) |
| Width | 12 |
| Information content | 13.2 |
| E-value | 1.3e-80 |
| Positional bias | 3.1e-23 |
| Strand bias | 0.46 |

| Distance scores for most similar known PSSMs | |
| --- | --- |
| S-000471 | 2.1 |
| S-000474 | 2.9 |
| S-000483 | 5.0 |
| M01238 | 9.3 |
| M00967 | 9.5 |


Cluster 14 Motif 3

| Motif Featuresmore... | |
| --- | --- |
| Sites | 110 (39.8%) |
| Width | 11 |
| Information content | 11.8 |
| E-value | 2.3e-16 |
| Positional bias | 0.0018 |
| Strand bias | 0.39 |

| Distance scores for most similar known PSSMs | |
| --- | --- |
| S-000460 | 7.9 |
| M01295 | 8.4 |
| M01136 | 8.7 |
| M00723 | 9.9 |
| S-000462 | 10.0 |


Cluster 14 Motif 4

| Motif Featuresmore... | |
| --- | --- |
| Sites | 46 (16.6%) |
| Width | 11 |
| Information content | 12.9 |
| E-value | 1e-07 |
| Positional bias | 0.002 |
| Strand bias | 0.44 |

| Distance scores for most similar known PSSMs | |
| --- | --- |
| RJH-00017 | 9.0 |
| S-000167 | 9.3 |
| S-000362 | 10.2 |
| MA-03491 | 11.0 |
| M01238 | 11.2 |


Cluster 14 Motif 5

| Motif Featuresmore... | |
| --- | --- |
| Sites | 115 (41.6%) |
| Width | 8 |
| Information content | 10.5 |
| E-value | 5e-17 |
| Positional bias | 1.3e-16 |
| Strand bias | 0.02 |

| Distance scores for most similar known PSSMs | |
| --- | --- |
| S-000474 | 0.5 |
| S-000471 | 3.4 |
| S-000483 | 4.0 |
| M01238 | 6.4 |
| M00967 | 6.9 |

  


Cluster 15 Motif 1

| Motif Featuresmore... | |
| --- | --- |
| Sites | 329 (71.9%) |
| Width | 12 |
| Information content | 14.2 |
| E-value | 8e-155 |
| Positional bias | 0.00019 |
| Strand bias | 0.25 |

| Distance scores for most similar known PSSMs | |
| --- | --- |
| S-000067 | 9.4 |
| HK-00008 | 10.6 |
| EVE-0008 | 10.6 |
| M01136 | 10.7 |


Cluster 15 Motif 2

| Motif Featuresmore... | |
| --- | --- |
| Sites | 162 (35.4%) |
| Width | 11 |
| Information content | 12.1 |
| E-value | 1.1e-71 |
| Positional bias | 1.7e-26 |
| Strand bias | 0.15 |

| Distance scores for most similar known PSSMs | |
| --- | --- |
| S-000474 | 1.7 |
| S-000471 | 2.2 |
| S-000483 | 4.4 |
| M01238 | 7.9 |
| M00967 | 8.0 |


Cluster 15 Motif 3

| Motif Featuresmore... | |
| --- | --- |
| Sites | 40 (8.7%) |
| Width | 12 |
| Information content | 15.8 |
| E-value | 1.6e-28 |
| Positional bias | 1.9e-08 |
| Strand bias | 0.13 |

| Distance scores for most similar known PSSMs | |
| --- | --- |
| S-000225 | 7.5 |
| S-000205 | 13.0 |
| MA-04111 | 14.0 |
| S-000402 | 14.2 |
| S-000131 | 14.3 |


Cluster 15 Motif 4

| Motif Featuresmore... | |
| --- | --- |
| Sites | 55 (12%) |
| Width | 11 |
| Information content | 13.8 |
| E-value | 7.5e-23 |
| Positional bias | 5.5e-08 |
| Strand bias | 0.3 |

| Distance scores for most similar known PSSMs | |
| --- | --- |
| RJH-00017 | 9.4 |
| S-000167 | 9.8 |
| S-000362 | 11.2 |
| S-000182 | 11.7 |
| MA-03491 | 11.9 |


Cluster 15 Motif 5

| Motif Featuresmore... | |
| --- | --- |
| Sites | 95 (20.7%) |
| Width | 11 |
| Information content | 12.9 |
| E-value | 1.4e-27 |
| Positional bias | 0.18 |
| Strand bias | 0.15 |

| Distance scores for most similar known PSSMs | |
| --- | --- |
| S-000159 | 9.0 |
| M00658 | 9.5 |
| M00046 | 12.0 |
| M01172 | 12.1 |
| M00029 | 12.5 |

  


Cluster 16 Motif 1

| Motif Featuresmore... | |
| --- | --- |
| Sites | 139 (85.8%) |
| Width | 12 |
| Information content | 13.8 |
| E-value | 4e-38 |
| Positional bias | 0.27 |
| Strand bias | 0.15 |

| Distance scores for most similar known PSSMs | |
| --- | --- |
| S-000067 | 8.5 |
| HK-00008 | 8.8 |
| EVE-0008 | 8.8 |
| S-000477 | 9.8 |


Cluster 16 Motif 2

| Motif Featuresmore... | |
| --- | --- |
| Sites | 68 (41.9%) |
| Width | 11 |
| Information content | 12.3 |
| E-value | 2.4e-18 |
| Positional bias | 0.00099 |
| Strand bias | 0.36 |

| Distance scores for most similar known PSSMs | |
| --- | --- |
| S-000460 | 8.5 |
| R02187 | 9.1 |
| MA-00391 | 9.9 |
| M01126 | 11.0 |
| MA-02051 | 11.3 |


Cluster 16 Motif 3

| Motif Featuresmore... | |
| --- | --- |
| Sites | 27 (16.6%) |
| Width | 11 |
| Information content | 11.2 |
| E-value | 0.19 |
| Positional bias | 0.21 |
| Strand bias | 0.5 |

| Distance scores for most similar known PSSMs | |
| --- | --- |
| MA-02831 | 8.4 |
| S-000417 | 9.3 |
| M01057 | 9.6 |
| MA-00792 | 9.8 |
| M01219 | 9.8 |


Cluster 16 Motif 4

| Motif Featuresmore... | |
| --- | --- |
| Sites | 33 (20.3%) |
| Width | 11 |
| Information content | 13.3 |
| E-value | 1.5 |
| Positional bias | 0.017 |
| Strand bias | 0.081 |

| Distance scores for most similar known PSSMs | |
| --- | --- |
| MA-00391 | 12.1 |
| S-000462 | 12.4 |
| S-000468 | 12.4 |
| S-000124 | 12.5 |
| M00028 | 12.7 |


Cluster 16 Motif 5

| Motif Featuresmore... | |
| --- | --- |
| Sites | 43 (26.5%) |
| Width | 7 |
| Information content | 10.9 |
| E-value | 20 |
| Positional bias | 9.5e-06 |
| Strand bias | 0.063 |

| Distance scores for most similar known PSSMs | |
| --- | --- |
| S-000474 | 1.6 |
| S-000483 | 5.5 |
| M00967 | 6.7 |
| M01238 | 7.3 |
| S-000115 | 8.1 |

  


  


Cluster 18 Motif 1

| Motif Featuresmore... | |
| --- | --- |
| Sites | 269 (93%) |
| Width | 12 |
| Information content | 12.9 |
| E-value | 8.6e-108 |
| Positional bias | 9.6e-05 |
| Strand bias | 0.025 |

| Distance scores for most similar known PSSMs | |
| --- | --- |
| R05016 | 9.3 |
| S-000067 | 9.5 |
| R02187 | 10.0 |
| M01126 | 10.2 |
| M01136 | 10.3 |


Cluster 18 Motif 2

| Motif Featuresmore... | |
| --- | --- |
| Sites | 88 (30.4%) |
| Width | 11 |
| Information content | 13.0 |
| E-value | 1.9e-37 |
| Positional bias | 2.6e-10 |
| Strand bias | 0.23 |

| Distance scores for most similar known PSSMs | |
| --- | --- |
| S-000474 | 2.8 |
| S-000471 | 3.1 |
| S-000483 | 6.2 |
| M01238 | 8.7 |
| M00967 | 9.2 |


Cluster 18 Motif 3

| Motif Featuresmore... | |
| --- | --- |
| Sites | 92 (31.8%) |
| Width | 11 |
| Information content | 12.1 |
| E-value | 8.6e-13 |
| Positional bias | 0.013 |
| Strand bias | 0.46 |

| Distance scores for most similar known PSSMs | |
| --- | --- |
| M01295 | 9.3 |
| M00658 | 9.6 |
| M01566 | 9.8 |
| MA-03771 | 10.0 |
| S-000159 | 10.3 |


Cluster 18 Motif 4

| Motif Featuresmore... | |
| --- | --- |
| Sites | 25 (8.6%) |
| Width | 12 |
| Information content | 16.0 |
| E-value | 6.5e-12 |
| Positional bias | 0.00044 |
| Strand bias | 0.11 |

| Distance scores for most similar known PSSMs | |
| --- | --- |
| S-000225 | 10.3 |
| S-000205 | 13.7 |
| R04577 | 14.1 |
| S-000131 | 14.4 |
| MA-04111 | 14.9 |


Cluster 18 Motif 5

| Motif Featuresmore... | |
| --- | --- |
| Sites | 27 (9.3%) |
| Width | 12 |
| Information content | 16.1 |
| E-value | 1.5e-09 |
| Positional bias | 0.016 |
| Strand bias | 0.22 |

| Distance scores for most similar known PSSMs | |
| --- | --- |
| RJH-00017 | 11.9 |
| S-000182 | 12.3 |
| S-000302 | 12.4 |
| MA-03491 | 12.4 |
| S-000167 | 13.1 |

  


Cluster 19 Motif 1

| Motif Featuresmore... | |
| --- | --- |
| Sites | 482 (78.2%) |
| Width | 12 |
| Information content | 14.3 |
| E-value | 1.9e-242 |
| Positional bias | 0.0019 |
| Strand bias | 0.45 |

| Distance scores for most similar known PSSMs | |
| --- | --- |
| S-000067 | 9.2 |
| MA-00491 | 9.8 |
| M00022 | 9.8 |
| R05016 | 9.9 |
| HK-00008 | 10.2 |


Cluster 19 Motif 2

| Motif Featuresmore... | |
| --- | --- |
| Sites | 202 (32.7%) |
| Width | 8 |
| Information content | 11.5 |
| E-value | 1.1e-106 |
| Positional bias | 1.6e-26 |
| Strand bias | 0.18 |

| Distance scores for most similar known PSSMs | |
| --- | --- |
| S-000474 | 2.1 |
| S-000483 | 4.5 |
| M00967 | 8.1 |
| M01238 | 8.4 |
| S-000216 | 8.7 |


Cluster 19 Motif 3

| Motif Featuresmore... | |
| --- | --- |
| Sites | 96 (15.5%) |
| Width | 12 |
| Information content | 14.5 |
| E-value | 5e-40 |
| Positional bias | 0.0026 |
| Strand bias | 0.13 |

| Distance scores for most similar known PSSMs | |
| --- | --- |
| S-000460 | 5.2 |
| M00723 | 9.4 |
| S-000427 | 10.5 |
| MA-02051 | 10.5 |
| R02187 | 10.6 |


Cluster 19 Motif 4

| Motif Featuresmore... | |
| --- | --- |
| Sites | 47 (7.6%) |
| Width | 12 |
| Information content | 15.3 |
| E-value | 3.8e-26 |
| Positional bias | 4.3e-06 |
| Strand bias | 0.0093 |

| Distance scores for most similar known PSSMs | |
| --- | --- |
| S-000225 | 7.9 |
| S-000205 | 12.5 |
| M01090 | 12.7 |
| R04577 | 13.4 |
| S-000131 | 13.5 |


Cluster 19 Motif 5

| Motif Featuresmore... | |
| --- | --- |
| Sites | 45 (7.3%) |
| Width | 12 |
| Information content | 14.7 |
| E-value | 7.9e-25 |
| Positional bias | 0.0063 |
| Strand bias | 0.068 |

| Distance scores for most similar known PSSMs | |
| --- | --- |
| RJH-00017 | 11.5 |
| S-000302 | 12.0 |
| M00110 | 12.0 |
| S-000362 | 12.5 |
| S-000182 | 13.2 |

  


Cluster 20 Motif 1

| Motif Featuresmore... | |
| --- | --- |
| Sites | 301 (57.5%) |
| Width | 12 |
| Information content | 15.2 |
| E-value | 1.6e-167 |
| Positional bias | 0.11 |
| Strand bias | 0.0019 |

| Distance scores for most similar known PSSMs | |
| --- | --- |
| HK-00008 | 8.8 |
| EVE-0008 | 8.8 |
| S-000067 | 9.3 |
| MA-00491 | 10.4 |


Cluster 20 Motif 2

| Motif Featuresmore... | |
| --- | --- |
| Sites | 226 (43.2%) |
| Width | 12 |
| Information content | 12.8 |
| E-value | 3.2e-140 |
| Positional bias | 7.4e-40 |
| Strand bias | 0.027 |

| Distance scores for most similar known PSSMs | |
| --- | --- |
| S-000474 | 2.0 |
| S-000471 | 2.2 |
| S-000483 | 4.8 |
| M01238 | 8.1 |
| M00967 | 8.1 |


Cluster 20 Motif 3

| Motif Featuresmore... | |
| --- | --- |
| Sites | 80 (15.2%) |
| Width | 12 |
| Information content | 13.6 |
| E-value | 2.1e-45 |
| Positional bias | 1.2e-08 |
| Strand bias | 0.11 |

| Distance scores for most similar known PSSMs | |
| --- | --- |
| S-000225 | 8.8 |
| S-000205 | 10.2 |
| S-000131 | 11.4 |
| M01057 | 11.5 |
| S-000497 | 12.0 |


Cluster 20 Motif 4

| Motif Featuresmore... | |
| --- | --- |
| Sites | 232 (44.3%) |
| Width | 12 |
| Information content | 12.0 |
| E-value | 2.5e-48 |
| Positional bias | 0.00026 |
| Strand bias | 0.13 |

| Distance scores for most similar known PSSMs | |
| --- | --- |
| S-000460 | 8.3 |
| MA-00391 | 8.9 |
| R02187 | 9.9 |
| MA-02051 | 10.0 |
| M01126 | 10.1 |


Cluster 20 Motif 5

| Motif Featuresmore... | |
| --- | --- |
| Sites | 112 (21.4%) |
| Width | 11 |
| Information content | 13.9 |
| E-value | 2.9e-18 |
| Positional bias | 0.079 |
| Strand bias | 0.054 |

| Distance scores for most similar known PSSMs | |
| --- | --- |
| S-000362 | 7.1 |
| S-000167 | 9.4 |
| S-000477 | 9.6 |
| S-000408 | 9.7 |
| S-000182 | 10.5 |

  


Cluster 21 Motif 1

| Motif Featuresmore... | |
| --- | --- |
| Sites | 59 (50.4%) |
| Width | 11 |
| Information content | 13.3 |
| E-value | 1.1e-45 |
| Positional bias | 2.7e-11 |
| Strand bias | 0.15 |

| Distance scores for most similar known PSSMs | |
| --- | --- |
| S-000471 | 2.6 |
| S-000474 | 3.2 |
| S-000483 | 5.3 |
| M01238 | 9.5 |
| S-000433 | 9.7 |


Cluster 21 Motif 2

| Motif Featuresmore... | |
| --- | --- |
| Sites | 117 (100%) |
| Width | 12 |
| Information content | 13.0 |
| E-value | 4.5e-29 |
| Positional bias | 0.031 |
| Strand bias | 0.098 |

| Distance scores for most similar known PSSMs | |
| --- | --- |
| M00972 | 8.2 |
| S-000067 | 8.3 |
| HK-00008 | 9.0 |
| EVE-0008 | 9.0 |


Cluster 21 Motif 3

| Motif Featuresmore... | |
| --- | --- |
| Sites | 56 (47.8%) |
| Width | 11 |
| Information content | 12.9 |
| E-value | 1.5e-05 |
| Positional bias | 1.1e-06 |
| Strand bias | 0.022 |

| Distance scores for most similar known PSSMs | |
| --- | --- |
| S-000308 | 2.5 |
| S-000472 | 3.0 |
| MA-04031 | 6.7 |
| M01493 | 7.4 |
| S-000115 | 7.5 |


Cluster 21 Motif 4

| Motif Featuresmore... | |
| --- | --- |
| Sites | 47 (40.1%) |
| Width | 8 |
| Information content | 11.1 |
| E-value | 3.9e-07 |
| Positional bias | 6.4e-12 |
| Strand bias | 0.39 |

| Distance scores for most similar known PSSMs | |
| --- | --- |
| S-000474 | 1.4 |
| S-000471 | 4.7 |
| S-000483 | 4.9 |
| M00967 | 7.3 |
| S-000115 | 8.2 |


Cluster 21 Motif 5

| Motif Featuresmore... | |
| --- | --- |
| Sites | 30 (25.6%) |
| Width | 11 |
| Information content | 13.0 |
| E-value | 28 |
| Positional bias | 0.0013 |
| Strand bias | 0.43 |

| Distance scores for most similar known PSSMs | |
| --- | --- |
| S-000115 | 10.2 |
| S-000362 | 10.3 |
| S-000182 | 10.7 |
| MA-00501 | 11.3 |
| S-000179 | 11.5 |

  


Cluster 22 Motif 1

| Motif Featuresmore... | |
| --- | --- |
| Sites | 274 (89.2%) |
| Width | 12 |
| Information content | 13.0 |
| E-value | 5e-103 |
| Positional bias | 2.9e-05 |
| Strand bias | 0.065 |

| Distance scores for most similar known PSSMs | |
| --- | --- |
| S-000067 | 8.5 |
| HK-00008 | 9.3 |
| EVE-0008 | 9.3 |
| M01126 | 9.6 |


Cluster 22 Motif 2

| Motif Featuresmore... | |
| --- | --- |
| Sites | 95 (30.9%) |
| Width | 11 |
| Information content | 13.4 |
| E-value | 1.7e-49 |
| Positional bias | 3e-19 |
| Strand bias | 0.42 |

| Distance scores for most similar known PSSMs | |
| --- | --- |
| S-000474 | 3.2 |
| S-000471 | 3.5 |
| S-000483 | 6.7 |
| M00967 | 8.9 |
| M01238 | 9.2 |


Cluster 22 Motif 3

| Motif Featuresmore... | |
| --- | --- |
| Sites | 76 (24.7%) |
| Width | 12 |
| Information content | 13.4 |
| E-value | 4.6e-20 |
| Positional bias | 1.1e-06 |
| Strand bias | 0.068 |

| Distance scores for most similar known PSSMs | |
| --- | --- |
| S-000362 | 9.7 |
| S-000182 | 10.7 |
| S-000167 | 11.2 |
| M01238 | 11.3 |
| M00486 | 11.5 |


Cluster 22 Motif 4

| Motif Featuresmore... | |
| --- | --- |
| Sites | 47 (15.3%) |
| Width | 11 |
| Information content | 14.1 |
| E-value | 8.1e-16 |
| Positional bias | 0.15 |
| Strand bias | 0.02 |

| Distance scores for most similar known PSSMs | |
| --- | --- |
| S-000460 | 5.1 |
| MA-02051 | 7.9 |
| R02187 | 8.8 |
| S-000486 | 10.9 |
| S-000427 | 11.5 |


Cluster 22 Motif 5

| Motif Featuresmore... | |
| --- | --- |
| Sites | 47 (15.3%) |
| Width | 12 |
| Information content | 11.8 |
| E-value | 8.6e-10 |
| Positional bias | 0.086 |
| Strand bias | 0.039 |

| Distance scores for most similar known PSSMs | |
| --- | --- |
| S-000205 | 8.2 |
| S-000146 | 8.7 |
| M01090 | 9.4 |
| S-000131 | 9.7 |
| M01057 | 10.2 |

  


Cluster 23 Motif 1

| Motif Featuresmore... | |
| --- | --- |
| Sites | 25 (96.1%) |
| Width | 12 |
| Information content | 15.4 |
| E-value | 0.004 |
| Positional bias | 0.23 |
| Strand bias | 0.00046 |

| Distance scores for most similar known PSSMs | |
| --- | --- |
| S-000067 | 8.0 |
| S-000477 | 10.2 |
| MA-00101 | 10.5 |
| S-000186 | 10.6 |
| HK-00008 | 10.8 |


Cluster 23 Motif 2

| Motif Featuresmore... | |
| --- | --- |
| Sites | 13 (50%) |
| Width | 12 |
| Information content | 11.2 |
| E-value | 78 |
| Positional bias | 0.61 |
| Strand bias | 0.5 |

| Distance scores for most similar known PSSMs | |
| --- | --- |
| M01057 | 10.2 |
| MA-02851 | 10.3 |
| MA-02131 | 10.5 |
| S-000482 | 10.5 |
| M01096 | 10.8 |


Cluster 23 Motif 3

| Motif Featuresmore... | |
| --- | --- |
| Sites | 14 (53.8%) |
| Width | 10 |
| Information content | 13.7 |
| E-value | 1.4e+02 |
| Positional bias | 0.84 |
| Strand bias | 0.6 |

| Distance scores for most similar known PSSMs | |
| --- | --- |
| R02180 | 10.5 |
| S-000143 | 10.6 |
| S-000353 | 11.3 |
| M00731 | 12.0 |
| M01131 | 12.1 |


Cluster 23 Motif 4

| Motif Featuresmore... | |
| --- | --- |
| Sites | 23 (88.4%) |
| Width | 12 |
| Information content | 12.7 |
| E-value | 1.4e+04 |
| Positional bias | 0.24 |
| Strand bias | 0.5 |

| Distance scores for most similar known PSSMs | |
| --- | --- |
| MA-02371 | 8.0 |
| R04592 | 8.9 |
| MA-00491 | 9.3 |
| M00022 | 9.3 |
| MA-03131 | 9.4 |


Cluster 23 Motif 5

| Motif Featuresmore... | |
| --- | --- |
| Sites | 3 (11.5%) |
| Width | 12 |
| Information content | 17.9 |
| E-value | 4.6e+04 |
| Positional bias | 0.61 |
| Strand bias | 0.5 |

| Distance scores for most similar known PSSMs | |
| --- | --- |
| M00531 | 14.0 |
| S-000478 | 15.0 |
| M00678 | 15.1 |
| MA-01441 | 16.9 |
| M00743 | 17.2 |

  


Cluster 24 Motif 1

| Motif Featuresmore... | |
| --- | --- |
| Sites | 56 (100%) |
| Width | 12 |
| Information content | 12.7 |
| E-value | 1.6e-09 |
| Positional bias | 0.22 |
| Strand bias | 0.011 |

| Distance scores for most similar known PSSMs | |
| --- | --- |
| HK-00008 | 6.9 |
| EVE-0008 | 6.9 |
| MA-00491 | 8.1 |
| M00022 | 8.1 |


Cluster 24 Motif 2

| Motif Featuresmore... | |
| --- | --- |
| Sites | 27 (48.2%) |
| Width | 12 |
| Information content | 10.8 |
| E-value | 0.013 |
| Positional bias | 0.0015 |
| Strand bias | 2.5e-05 |

| Distance scores for most similar known PSSMs | |
| --- | --- |
| MA-02051 | 9.7 |
| M00807 | 9.9 |
| S-000499 | 10.6 |
| M01240 | 11.1 |
| S-000462 | 11.2 |


Cluster 24 Motif 3

| Motif Featuresmore... | |
| --- | --- |
| Sites | 25 (44.6%) |
| Width | 10 |
| Information content | 12.4 |
| E-value | 2e+04 |
| Positional bias | 0.45 |
| Strand bias | 0.5 |

| Distance scores for most similar known PSSMs | |
| --- | --- |
| S-000142 | 4.6 |
| S-000310 | 6.3 |
| RJH-00021 | 7.4 |
| S-000390 | 7.5 |
| S-000457 | 8.2 |


Cluster 24 Motif 4

| Motif Featuresmore... | |
| --- | --- |
| Sites | 2 (3.5%) |
| Width | 11 |
| Information content | 17.6 |
| E-value | 1.3e+05 |
| Positional bias | 0.69 |
| Strand bias | 0.25 |

| Distance scores for most similar known PSSMs | |
| --- | --- |
| M01303 | 14.0 |
| RJH-00015 | 14.4 |
| S-000179 | 14.4 |
| R02121 | 15.0 |
| S-000474 | 15.7 |


Cluster 24 Motif 5

| Motif Featuresmore... | |
| --- | --- |
| Sites | 2 (3.5%) |
| Width | 11 |
| Information content | 17.6 |
| E-value | 1.5e+05 |
| Positional bias | 0.15 |
| Strand bias | 0.75 |

| Distance scores for most similar known PSSMs | |
| --- | --- |
| M01113 | 13.5 |
| MA-02681 | 14.6 |
| R02121 | 14.9 |
| R05065 | 14.9 |
| M00479 | 14.9 |

  


Cluster 25 Motif 1

| Motif Featuresmore... | |
| --- | --- |
| Sites | 71 (100%) |
| Width | 12 |
| Information content | 14.1 |
| E-value | 1.1e-29 |
| Positional bias | 0.82 |
| Strand bias | 0.5 |

| Distance scores for most similar known PSSMs | |
| --- | --- |
| MA-02771 | 8.8 |
| M01136 | 9.0 |
| M00972 | 9.1 |
| S-000067 | 9.7 |
| HK-00008 | 10.0 |


Cluster 25 Motif 2

| Motif Featuresmore... | |
| --- | --- |
| Sites | 20 (28.1%) |
| Width | 12 |
| Information content | 12.5 |
| E-value | 0.35 |
| Positional bias | 0.038 |
| Strand bias | 0.59 |

| Distance scores for most similar known PSSMs | |
| --- | --- |
| S-000460 | 9.2 |
| MA-02051 | 9.9 |
| M00723 | 10.0 |
| M00649 | 11.0 |
| MA-00391 | 11.7 |


Cluster 25 Motif 3

| Motif Featuresmore... | |
| --- | --- |
| Sites | 13 (18.3%) |
| Width | 12 |
| Information content | 12.7 |
| E-value | 86 |
| Positional bias | 0.41 |
| Strand bias | 0.29 |

| Distance scores for most similar known PSSMs | |
| --- | --- |
| M00046 | 12.0 |
| M00948 | 12.4 |
| M01100 | 13.2 |
| MA-01301 | 13.5 |
| M01160 | 13.6 |


Cluster 25 Motif 4

| Motif Featuresmore... | |
| --- | --- |
| Sites | 33 (46.4%) |
| Width | 11 |
| Information content | 13.2 |
| E-value | 2.9e+03 |
| Positional bias | 0.0076 |
| Strand bias | 0.24 |

| Distance scores for most similar known PSSMs | |
| --- | --- |
| M00438 | 8.2 |
| M01021 | 8.5 |
| MA-02051 | 8.6 |
| M01126 | 9.8 |
| M01136 | 9.8 |


Cluster 25 Motif 5

| Motif Featuresmore... | |
| --- | --- |
| Sites | 13 (18.3%) |
| Width | 12 |
| Information content | 13.2 |
| E-value | 3.1e+03 |
| Positional bias | 0.94 |
| Strand bias | 0.13 |

| Distance scores for most similar known PSSMs | |
| --- | --- |
| M00807 | 12.1 |
| M01113 | 13.0 |
| M00769 | 13.2 |
| M00984 | 13.2 |
| R02251 | 13.3 |

  


Cluster 26 Motif 1

| Motif Featuresmore... | |
| --- | --- |
| Sites | 108 (80.5%) |
| Width | 12 |
| Information content | 14.0 |
| E-value | 3.6e-39 |
| Positional bias | 0.83 |
| Strand bias | 0.25 |

| Distance scores for most similar known PSSMs | |
| --- | --- |
| S-000067 | 10.6 |
| MA-00451 | 10.9 |
| M01136 | 11.0 |
| HK-00008 | 11.4 |
| EVE-0008 | 11.4 |


Cluster 26 Motif 2

| Motif Featuresmore... | |
| --- | --- |
| Sites | 30 (22.3%) |
| Width | 11 |
| Information content | 13.8 |
| E-value | 1.9e-06 |
| Positional bias | 0.0011 |
| Strand bias | 0.18 |

| Distance scores for most similar known PSSMs | |
| --- | --- |
| M01126 | 11.4 |
| S-000424 | 11.4 |
| S-000124 | 12.3 |
| S-000462 | 12.4 |
| S-000468 | 12.4 |


Cluster 26 Motif 3

| Motif Featuresmore... | |
| --- | --- |
| Sites | 58 (43.2%) |
| Width | 8 |
| Information content | 10.9 |
| E-value | 0.13 |
| Positional bias | 0.27 |
| Strand bias | 0.0027 |

| Distance scores for most similar known PSSMs | |
| --- | --- |
| RJH-00017 | 7.7 |
| MA-01331 | 8.9 |
| M00456 | 8.9 |
| M01092 | 9.2 |
| M01030 | 9.4 |


Cluster 26 Motif 4

| Motif Featuresmore... | |
| --- | --- |
| Sites | 26 (19.4%) |
| Width | 10 |
| Information content | 11.7 |
| E-value | 90 |
| Positional bias | 0.073 |
| Strand bias | 0.16 |

| Distance scores for most similar known PSSMs | |
| --- | --- |
| M01585 | 2.4 |
| M00055 | 3.1 |
| MA-01041 | 3.8 |
| R04649 | 3.9 |
| S-000042 | 3.9 |


Cluster 26 Motif 5

| Motif Featuresmore... | |
| --- | --- |
| Sites | 8 (5.9%) |
| Width | 12 |
| Information content | 13.3 |
| E-value | 4.7e+03 |
| Positional bias | 0.25 |
| Strand bias | 0.14 |

| Distance scores for most similar known PSSMs | |
| --- | --- |
| R02148 | 8.3 |
| M00982 | 10.1 |
| MA-01621 | 10.4 |
| R02262 | 11.1 |
| M01219 | 11.7 |

  


Cluster 27 Motif 1

| Motif Featuresmore... | |
| --- | --- |
| Sites | 178 (91.2%) |
| Width | 12 |
| Information content | 13.4 |
| E-value | 8.1e-59 |
| Positional bias | 0.012 |
| Strand bias | 0.3 |

| Distance scores for most similar known PSSMs | |
| --- | --- |
| S-000067 | 8.4 |
| HK-00008 | 8.6 |
| EVE-0008 | 8.6 |
| MA-02771 | 9.7 |


Cluster 27 Motif 2

| Motif Featuresmore... | |
| --- | --- |
| Sites | 28 (14.3%) |
| Width | 11 |
| Information content | 12.3 |
| E-value | 0.79 |
| Positional bias | 0.0034 |
| Strand bias | 0.57 |

| Distance scores for most similar known PSSMs | |
| --- | --- |
| M00942 | 3.9 |
| S-000041 | 4.1 |
| M01116 | 4.2 |
| M00368 | 4.2 |
| M00055 | 4.4 |


Cluster 27 Motif 3

| Motif Featuresmore... | |
| --- | --- |
| Sites | 116 (59.4%) |
| Width | 12 |
| Information content | 11.1 |
| E-value | 0.0028 |
| Positional bias | 0.0021 |
| Strand bias | 0.11 |

| Distance scores for most similar known PSSMs | |
| --- | --- |
| M01126 | 8.9 |
| MA-00391 | 9.2 |
| M00723 | 10.0 |
| S-000245 | 10.5 |
| M00500 | 10.6 |


Cluster 27 Motif 4

| Motif Featuresmore... | |
| --- | --- |
| Sites | 26 (13.3%) |
| Width | 12 |
| Information content | 13.1 |
| E-value | 98 |
| Positional bias | 0.021 |
| Strand bias | 0.014 |

| Distance scores for most similar known PSSMs | |
| --- | --- |
| M00257 | 10.5 |
| MA-01331 | 11.8 |
| M00378 | 12.2 |
| M00633 | 12.3 |
| M01082 | 12.5 |


Cluster 27 Motif 5

| Motif Featuresmore... | |
| --- | --- |
| Sites | 12 (6.1%) |
| Width | 12 |
| Information content | 14.7 |
| E-value | 3.3e+03 |
| Positional bias | 0.15 |
| Strand bias | 0.61 |

| Distance scores for most similar known PSSMs | |
| --- | --- |
| S-000205 | 10.5 |
| S-000131 | 11.4 |
| MA-00182 | 13.9 |
| R02126 | 14.0 |
| M00443 | 14.0 |

  


Cluster 28 Motif 1

| Motif Featuresmore... | |
| --- | --- |
| Sites | 174 (67.7%) |
| Width | 12 |
| Information content | 15.3 |
| E-value | 1.8e-82 |
| Positional bias | 0.55 |
| Strand bias | 0.056 |

| Distance scores for most similar known PSSMs | |
| --- | --- |
| S-000067 | 9.6 |
| HK-00008 | 9.9 |
| EVE-0008 | 9.9 |
| MA-00101 | 11.0 |


Cluster 28 Motif 2

| Motif Featuresmore... | |
| --- | --- |
| Sites | 47 (18.2%) |
| Width | 11 |
| Information content | 14.0 |
| E-value | 1.6e-11 |
| Positional bias | 0.00061 |
| Strand bias | 0.02 |

| Distance scores for most similar known PSSMs | |
| --- | --- |
| M00658 | 8.9 |
| M01172 | 10.6 |
| MA-01271 | 11.0 |
| S-000344 | 11.2 |
| M00746 | 11.2 |


Cluster 28 Motif 3

| Motif Featuresmore... | |
| --- | --- |
| Sites | 58 (22.5%) |
| Width | 12 |
| Information content | 13.1 |
| E-value | 7.5e-08 |
| Positional bias | 2.2e-10 |
| Strand bias | 0.26 |

| Distance scores for most similar known PSSMs | |
| --- | --- |
| S-000460 | 4.9 |
| MA-02051 | 7.3 |
| M00723 | 8.5 |
| S-000462 | 10.2 |
| S-000468 | 10.2 |


Cluster 28 Motif 4

| Motif Featuresmore... | |
| --- | --- |
| Sites | 170 (66.1%) |
| Width | 12 |
| Information content | 12.3 |
| E-value | 3.6e-12 |
| Positional bias | 0.65 |
| Strand bias | 0.039 |

| Distance scores for most similar known PSSMs | |
| --- | --- |
| S-000477 | 8.6 |
| M01252 | 8.6 |
| HK-00008 | 9.5 |
| EVE-0008 | 9.5 |


Cluster 28 Motif 5

| Motif Featuresmore... | |
| --- | --- |
| Sites | 17 (6.6%) |
| Width | 11 |
| Information content | 14.9 |
| E-value | 0.39 |
| Positional bias | 0.0034 |
| Strand bias | 0.5 |

| Distance scores for most similar known PSSMs | |
| --- | --- |
| S-000041 | 2.3 |
| S-000119 | 3.6 |
| M00368 | 4.3 |
| M00942 | 4.8 |
| S-000505 | 5.1 |

  


Cluster 29 Motif 1

| Motif Featuresmore... | |
| --- | --- |
| Sites | 204 (67.5%) |
| Width | 12 |
| Information content | 13.7 |
| E-value | 4.4e-88 |
| Positional bias | 0.0018 |
| Strand bias | 0.36 |

| Distance scores for most similar known PSSMs | |
| --- | --- |
| R05016 | 10.3 |
| M00972 | 10.3 |
| M01126 | 10.8 |
| M01247 | 11.0 |
| R02187 | 11.0 |


Cluster 29 Motif 2

| Motif Featuresmore... | |
| --- | --- |
| Sites | 57 (18.8%) |
| Width | 11 |
| Information content | 13.7 |
| E-value | 1.5e-24 |
| Positional bias | 1.7e-06 |
| Strand bias | 0.0038 |

| Distance scores for most similar known PSSMs | |
| --- | --- |
| S-000460 | 5.0 |
| MA-02051 | 9.5 |
| S-000391 | 11.1 |
| S-000427 | 11.8 |
| S-000270 | 12.0 |


Cluster 29 Motif 3

| Motif Featuresmore... | |
| --- | --- |
| Sites | 218 (72.1%) |
| Width | 12 |
| Information content | 13.2 |
| E-value | 2e-24 |
| Positional bias | 0.6 |
| Strand bias | 0.0007 |

| Distance scores for most similar known PSSMs | |
| --- | --- |
| S-000477 | 6.9 |
| S-000067 | 8.3 |
| M01216 | 8.7 |
| HK-00008 | 8.9 |
| EVE-0008 | 8.9 |


Cluster 29 Motif 4

| Motif Featuresmore... | |
| --- | --- |
| Sites | 57 (18.8%) |
| Width | 7 |
| Information content | 11.3 |
| E-value | 9.5e-12 |
| Positional bias | 0.0001 |
| Strand bias | 0.3 |

| Distance scores for most similar known PSSMs | |
| --- | --- |
| S-000471 | 1.6 |
| S-000474 | 2.2 |
| S-000483 | 2.7 |
| MA-03811 | 7.0 |
| S-000216 | 7.1 |


Cluster 29 Motif 5

| Motif Featuresmore... | |
| --- | --- |
| Sites | 35 (11.5%) |
| Width | 11 |
| Information content | 12.8 |
| E-value | 3.4e-06 |
| Positional bias | 0.053 |
| Strand bias | 0.37 |

| Distance scores for most similar known PSSMs | |
| --- | --- |
| S-000205 | 10.4 |
| M01057 | 11.0 |
| S-000131 | 11.2 |
| S-000418 | 11.9 |
| R04577 | 12.0 |

  


Cluster 30 Motif 1

| Motif Featuresmore... | |
| --- | --- |
| Sites | 208 (80.3%) |
| Width | 12 |
| Information content | 13.3 |
| E-value | 1.8e-67 |
| Positional bias | 0.029 |
| Strand bias | 0.36 |

| Distance scores for most similar known PSSMs | |
| --- | --- |
| R05016 | 9.3 |
| S-000477 | 9.6 |
| M01216 | 10.1 |
| HK-00008 | 10.1 |
| EVE-0008 | 10.1 |


Cluster 30 Motif 2

| Motif Featuresmore... | |
| --- | --- |
| Sites | 40 (15.4%) |
| Width | 8 |
| Information content | 11.6 |
| E-value | 0.004 |
| Positional bias | 0.44 |
| Strand bias | 0.44 |

| Distance scores for most similar known PSSMs | |
| --- | --- |
| S-000471 | 1.5 |
| S-000474 | 2.6 |
| S-000483 | 3.1 |
| MA-03811 | 7.4 |
| S-000216 | 7.4 |


Cluster 30 Motif 3

| Motif Featuresmore... | |
| --- | --- |
| Sites | 26 (10%) |
| Width | 8 |
| Information content | 12.5 |
| E-value | 0.0073 |
| Positional bias | 0.0026 |
| Strand bias | 0.16 |

| Distance scores for most similar known PSSMs | |
| --- | --- |
| S-000119 | 1.5 |
| M00366 | 1.6 |
| S-000394 | 2.3 |
| MA-03101 | 2.4 |
| M01584 | 2.6 |


Cluster 30 Motif 4

| Motif Featuresmore... | |
| --- | --- |
| Sites | 46 (17.7%) |
| Width | 11 |
| Information content | 12.3 |
| E-value | 0.054 |
| Positional bias | 0.0042 |
| Strand bias | 0.052 |

| Distance scores for most similar known PSSMs | |
| --- | --- |
| MA-02871 | 9.0 |
| M00658 | 9.3 |
| MA-02051 | 10.1 |
| MA-00391 | 10.9 |
| R02232 | 11.1 |


Cluster 30 Motif 5

| Motif Featuresmore... | |
| --- | --- |
| Sites | 78 (30.1%) |
| Width | 11 |
| Information content | 13.5 |
| E-value | 0.0011 |
| Positional bias | 0.98 |
| Strand bias | 0.044 |

| Distance scores for most similar known PSSMs | |
| --- | --- |
| HK-00008 | 8.2 |
| EVE-0008 | 8.2 |
| R02164 | 10.2 |
| R04693 | 10.6 |

  


Cluster 31 Motif 1

| Motif Featuresmore... | |
| --- | --- |
| Sites | 174 (75.3%) |
| Width | 12 |
| Information content | 14.2 |
| E-value | 2.3e-65 |
| Positional bias | 0.07 |
| Strand bias | 0.04 |

| Distance scores for most similar known PSSMs | |
| --- | --- |
| S-000067 | 9.3 |
| M00274 | 9.6 |
| HK-00008 | 9.9 |
| EVE-0008 | 9.9 |


Cluster 31 Motif 2

| Motif Featuresmore... | |
| --- | --- |
| Sites | 70 (30.3%) |
| Width | 11 |
| Information content | 13.5 |
| E-value | 2.8e-25 |
| Positional bias | 1e-06 |
| Strand bias | 0.06 |

| Distance scores for most similar known PSSMs | |
| --- | --- |
| S-000473 | 7.9 |
| S-000159 | 8.4 |
| M00658 | 9.1 |
| M01172 | 9.8 |
| R02232 | 10.6 |


Cluster 31 Motif 3

| Motif Featuresmore... | |
| --- | --- |
| Sites | 48 (20.7%) |
| Width | 12 |
| Information content | 13.7 |
| E-value | 9.3e-19 |
| Positional bias | 0.0023 |
| Strand bias | 0.03 |

| Distance scores for most similar known PSSMs | |
| --- | --- |
| S-000460 | 5.3 |
| MA-02051 | 7.8 |
| M00723 | 7.9 |
| S-000391 | 9.5 |
| M00438 | 9.8 |


Cluster 31 Motif 4

| Motif Featuresmore... | |
| --- | --- |
| Sites | 33 (14.2%) |
| Width | 12 |
| Information content | 15.9 |
| E-value | 6.1e-11 |
| Positional bias | 0.056 |
| Strand bias | 0.04 |

| Distance scores for most similar known PSSMs | |
| --- | --- |
| S-000362 | 10.8 |
| S-000353 | 12.6 |
| RJH-00017 | 12.7 |
| M01082 | 12.8 |
| S-000167 | 12.9 |


Cluster 31 Motif 5

| Motif Featuresmore... | |
| --- | --- |
| Sites | 17 (7.3%) |
| Width | 11 |
| Information content | 14.1 |
| E-value | 14 |
| Positional bias | 0.052 |
| Strand bias | 0.17 |

| Distance scores for most similar known PSSMs | |
| --- | --- |
| M00632 | 12.9 |
| MA-02051 | 12.9 |
| M00695 | 13.0 |
| S-000369 | 13.6 |
| M00084 | 14.0 |

  


Cluster 32 Motif 1

| Motif Featuresmore... | |
| --- | --- |
| Sites | 111 (73.5%) |
| Width | 12 |
| Information content | 14.2 |
| E-value | 4.8e-41 |
| Positional bias | 0.0016 |
| Strand bias | 0.42 |

| Distance scores for most similar known PSSMs | |
| --- | --- |
| HK-00008 | 10.4 |
| EVE-0008 | 10.4 |
| S-000067 | 10.5 |
| MA-00491 | 10.8 |


Cluster 32 Motif 2

| Motif Featuresmore... | |
| --- | --- |
| Sites | 46 (30.4%) |
| Width | 11 |
| Information content | 11.9 |
| E-value | 6.4e-09 |
| Positional bias | 0.0024 |
| Strand bias | 0.23 |

| Distance scores for most similar known PSSMs | |
| --- | --- |
| MA-02051 | 7.0 |
| S-000460 | 7.3 |
| MA-00391 | 9.3 |
| R02187 | 9.9 |
| R02137 | 10.2 |


Cluster 32 Motif 3

| Motif Featuresmore... | |
| --- | --- |
| Sites | 64 (42.3%) |
| Width | 12 |
| Information content | 13.7 |
| E-value | 0.00076 |
| Positional bias | 0.45 |
| Strand bias | 0.084 |

| Distance scores for most similar known PSSMs | |
| --- | --- |
| R02164 | 6.4 |
| S-000362 | 9.1 |
| HK-00008 | 9.3 |
| EVE-0008 | 9.3 |


Cluster 32 Motif 4

| Motif Featuresmore... | |
| --- | --- |
| Sites | 28 (18.5%) |
| Width | 6 |
| Information content | 10.6 |
| E-value | 37 |
| Positional bias | 0.88 |
| Strand bias | 0.57 |

| Distance scores for most similar known PSSMs | |
| --- | --- |
| S-000474 | 1.7 |
| S-000471 | 1.9 |
| S-000483 | 2.1 |
| M00948 | 6.2 |
| MA-03811 | 6.4 |


Cluster 32 Motif 5

| Motif Featuresmore... | |
| --- | --- |
| Sites | 16 (10.5%) |
| Width | 11 |
| Information content | 14.4 |
| E-value | 44 |
| Positional bias | 0.0002 |
| Strand bias | 0.038 |

| Distance scores for most similar known PSSMs | |
| --- | --- |
| S-000167 | 9.8 |
| RJH-00017 | 10.4 |
| S-000362 | 11.4 |
| M00226 | 12.3 |
| S-000182 | 12.5 |

  


Cluster 33 Motif 1

| Motif Featuresmore... | |
| --- | --- |
| Sites | 176 (82.6%) |
| Width | 12 |
| Information content | 14.2 |
| E-value | 1.2e-65 |
| Positional bias | 0.29 |
| Strand bias | 0.0096 |

| Distance scores for most similar known PSSMs | |
| --- | --- |
| S-000067 | 8.8 |
| MA-00491 | 10.0 |
| M00022 | 10.0 |
| HK-00008 | 10.1 |
| EVE-0008 | 10.1 |


Cluster 33 Motif 2

| Motif Featuresmore... | |
| --- | --- |
| Sites | 25 (11.7%) |
| Width | 10 |
| Information content | 13.3 |
| E-value | 2.5e-07 |
| Positional bias | 0.014 |
| Strand bias | 0.054 |

| Distance scores for most similar known PSSMs | |
| --- | --- |
| S-000041 | 2.3 |
| S-000119 | 3.1 |
| M00368 | 3.7 |
| M00367 | 3.8 |
| M01584 | 4.0 |


Cluster 33 Motif 3

| Motif Featuresmore... | |
| --- | --- |
| Sites | 59 (27.6%) |
| Width | 12 |
| Information content | 12.4 |
| E-value | 5.8e-05 |
| Positional bias | 9.8e-05 |
| Strand bias | 0.15 |

| Distance scores for most similar known PSSMs | |
| --- | --- |
| S-000460 | 7.8 |
| MA-02051 | 7.9 |
| M00723 | 9.3 |
| R02187 | 10.4 |
| S-000270 | 10.5 |


Cluster 33 Motif 4

| Motif Featuresmore... | |
| --- | --- |
| Sites | 64 (30%) |
| Width | 11 |
| Information content | 13.7 |
| E-value | 2.5 |
| Positional bias | 0.13 |
| Strand bias | 0.0018 |

| Distance scores for most similar known PSSMs | |
| --- | --- |
| S-000308 | 4.7 |
| S-000472 | 5.1 |
| M00486 | 9.1 |
| S-000115 | 9.2 |
| S-000127 | 9.6 |


Cluster 33 Motif 5

| Motif Featuresmore... | |
| --- | --- |
| Sites | 11 (5.1%) |
| Width | 11 |
| Information content | 13.9 |
| E-value | 7.7e+02 |
| Positional bias | 0.023 |
| Strand bias | 0.033 |

| Distance scores for most similar known PSSMs | |
| --- | --- |
| MA-04041 | 10.7 |
| MA-02761 | 11.5 |
| R02148 | 12.7 |
| M00306 | 12.8 |
| S-000153 | 13.3 |

  


Cluster 34 Motif 1

| Motif Featuresmore... | |
| --- | --- |
| Sites | 91 (100%) |
| Width | 12 |
| Information content | 13.6 |
| E-value | 1.2e-31 |
| Positional bias | 0.93 |
| Strand bias | 0.26 |

| Distance scores for most similar known PSSMs | |
| --- | --- |
| MA-02771 | 8.3 |
| S-000067 | 8.6 |
| M01136 | 9.1 |
| HK-00008 | 9.7 |
| EVE-0008 | 9.7 |


Cluster 34 Motif 2

| Motif Featuresmore... | |
| --- | --- |
| Sites | 58 (63.7%) |
| Width | 11 |
| Information content | 11.5 |
| E-value | 0.051 |
| Positional bias | 0.32 |
| Strand bias | 0.45 |

| Distance scores for most similar known PSSMs | |
| --- | --- |
| MA-02051 | 6.8 |
| M01136 | 8.5 |
| M01126 | 9.1 |
| MA-00391 | 9.4 |
| S-000462 | 9.4 |


Cluster 34 Motif 3

| Motif Featuresmore... | |
| --- | --- |
| Sites | 19 (20.8%) |
| Width | 10 |
| Information content | 12.4 |
| E-value | 0.16 |
| Positional bias | 0.88 |
| Strand bias | 0.032 |

| Distance scores for most similar known PSSMs | |
| --- | --- |
| M00367 | 3.2 |
| S-000041 | 3.3 |
| M00368 | 3.5 |
| M00369 | 3.6 |
| S-000287 | 3.8 |


Cluster 34 Motif 4

| Motif Featuresmore... | |
| --- | --- |
| Sites | 10 (10.9%) |
| Width | 11 |
| Information content | 15.5 |
| E-value | 9.3e+02 |
| Positional bias | 0.1 |
| Strand bias | 0.62 |

| Distance scores for most similar known PSSMs | |
| --- | --- |
| S-000205 | 10.8 |
| S-000131 | 11.6 |
| R02126 | 12.0 |
| MA-04111 | 12.0 |
| S-000225 | 12.5 |


Cluster 34 Motif 5

| Motif Featuresmore... | |
| --- | --- |
| Sites | 13 (14.2%) |
| Width | 12 |
| Information content | 15.8 |
| E-value | 0.86 |
| Positional bias | 0.18 |
| Strand bias | 0.5 |

| Distance scores for most similar known PSSMs | |
| --- | --- |
| MA-03591 | 11.7 |
| S-000143 | 13.7 |
| M00213 | 14.3 |
| S-000148 | 14.5 |
| M01156 | 14.7 |

  


  


Cluster 36 Motif 1

| Motif Featuresmore... | |
| --- | --- |
| Sites | 182 (75.2%) |
| Width | 12 |
| Information content | 15.3 |
| E-value | 2.2e-109 |
| Positional bias | 0.59 |
| Strand bias | 0.13 |

| Distance scores for most similar known PSSMs | |
| --- | --- |
| HK-00008 | 8.6 |
| EVE-0008 | 8.6 |
| S-000067 | 9.1 |
| MA-00491 | 10.4 |


Cluster 36 Motif 2

| Motif Featuresmore... | |
| --- | --- |
| Sites | 131 (54.1%) |
| Width | 11 |
| Information content | 11.7 |
| E-value | 8.9e-36 |
| Positional bias | 0.002 |
| Strand bias | 0.11 |

| Distance scores for most similar known PSSMs | |
| --- | --- |
| S-000460 | 7.8 |
| MA-02051 | 9.2 |
| R02187 | 9.3 |
| M01126 | 9.7 |
| S-000245 | 9.8 |


Cluster 36 Motif 3

| Motif Featuresmore... | |
| --- | --- |
| Sites | 17 (7%) |
| Width | 11 |
| Information content | 15.7 |
| E-value | 0.036 |
| Positional bias | 0.52 |
| Strand bias | 0.31 |

| Distance scores for most similar known PSSMs | |
| --- | --- |
| S-000205 | 11.0 |
| S-000225 | 11.1 |
| S-000121 | 11.9 |
| S-000131 | 13.3 |
| R04588 | 13.4 |


Cluster 36 Motif 4

| Motif Featuresmore... | |
| --- | --- |
| Sites | 12 (4.9%) |
| Width | 11 |
| Information content | 13.1 |
| E-value | 4.2e+03 |
| Positional bias | 0.46 |
| Strand bias | 0.61 |

| Distance scores for most similar known PSSMs | |
| --- | --- |
| M00067 | 9.8 |
| S-000291 | 10.1 |
| S-000507 | 10.2 |
| RJH-00010 | 10.3 |
| M01585 | 10.6 |


Cluster 36 Motif 5

| Motif Featuresmore... | |
| --- | --- |
| Sites | 30 (12.3%) |
| Width | 8 |
| Information content | 12.3 |
| E-value | 8.5e+04 |
| Positional bias | 0.46 |
| Strand bias | 0.43 |

| Distance scores for most similar known PSSMs | |
| --- | --- |
| S-000474 | 3.3 |
| S-000471 | 6.7 |
| S-000483 | 6.9 |
| M00967 | 7.6 |
| MA-00201 | 9.7 |

  


Cluster 37 Motif 1

| Motif Featuresmore... | |
| --- | --- |
| Sites | 209 (78.8%) |
| Width | 12 |
| Information content | 14.8 |
| E-value | 1.8e-113 |
| Positional bias | 0.0045 |
| Strand bias | 0.2 |

| Distance scores for most similar known PSSMs | |
| --- | --- |
| S-000067 | 9.3 |
| HK-00008 | 10.2 |
| EVE-0008 | 10.2 |
| S-000477 | 10.6 |


Cluster 37 Motif 2

| Motif Featuresmore... | |
| --- | --- |
| Sites | 71 (26.7%) |
| Width | 8 |
| Information content | 11.8 |
| E-value | 1.1e-32 |
| Positional bias | 2e-10 |
| Strand bias | 0.12 |

| Distance scores for most similar known PSSMs | |
| --- | --- |
| S-000474 | 2.5 |
| S-000483 | 3.0 |
| MA-03811 | 7.3 |
| S-000216 | 8.3 |
| S-000026 | 9.2 |


Cluster 37 Motif 3

| Motif Featuresmore... | |
| --- | --- |
| Sites | 113 (42.6%) |
| Width | 11 |
| Information content | 12.6 |
| E-value | 3.5e-26 |
| Positional bias | 0.1 |
| Strand bias | 0.43 |

| Distance scores for most similar known PSSMs | |
| --- | --- |
| M01295 | 7.6 |
| S-000460 | 7.7 |
| M01136 | 8.4 |
| M01126 | 10.0 |
| M01172 | 10.3 |


Cluster 37 Motif 4

| Motif Featuresmore... | |
| --- | --- |
| Sites | 29 (10.9%) |
| Width | 10 |
| Information content | 13.3 |
| E-value | 8.5e-07 |
| Positional bias | 0.0047 |
| Strand bias | 0.23 |

| Distance scores for most similar known PSSMs | |
| --- | --- |
| S-000041 | 2.8 |
| S-000406 | 3.0 |
| S-000119 | 3.2 |
| RJH-00009 | 3.7 |
| M00368 | 3.9 |


Cluster 37 Motif 5

| Motif Featuresmore... | |
| --- | --- |
| Sites | 43 (16.2%) |
| Width | 11 |
| Information content | 14.7 |
| E-value | 4.6e-10 |
| Positional bias | 0.054 |
| Strand bias | 0.5 |

| Distance scores for most similar known PSSMs | |
| --- | --- |
| RJH-00017 | 9.1 |
| S-000408 | 10.2 |
| S-000362 | 10.5 |
| S-000167 | 10.8 |
| S-000182 | 11.1 |

  


Cluster 38 Motif 1

| Motif Featuresmore... | |
| --- | --- |
| Sites | 321 (98.4%) |
| Width | 12 |
| Information content | 13.1 |
| E-value | 4.5e-109 |
| Positional bias | 0.2 |
| Strand bias | 0.0036 |

| Distance scores for most similar known PSSMs | |
| --- | --- |
| S-000067 | 8.5 |
| MA-01201 | 8.6 |
| HK-00008 | 8.9 |
| EVE-0008 | 8.9 |


Cluster 38 Motif 2

| Motif Featuresmore... | |
| --- | --- |
| Sites | 90 (27.6%) |
| Width | 8 |
| Information content | 11.8 |
| E-value | 9.6e-34 |
| Positional bias | 1.3e-12 |
| Strand bias | 0.23 |

| Distance scores for most similar known PSSMs | |
| --- | --- |
| S-000474 | 2.2 |
| S-000471 | 4.8 |
| S-000483 | 4.9 |
| M00967 | 7.2 |
| M00948 | 9.5 |


Cluster 38 Motif 3

| Motif Featuresmore... | |
| --- | --- |
| Sites | 108 (33.1%) |
| Width | 12 |
| Information content | 12.3 |
| E-value | 3.2e-31 |
| Positional bias | 0.1 |
| Strand bias | 0.32 |

| Distance scores for most similar known PSSMs | |
| --- | --- |
| MA-02051 | 5.6 |
| S-000460 | 5.9 |
| M00723 | 7.1 |
| S-000270 | 9.9 |
| M00438 | 10.0 |


Cluster 38 Motif 4

| Motif Featuresmore... | |
| --- | --- |
| Sites | 19 (5.8%) |
| Width | 12 |
| Information content | 14.8 |
| E-value | 1.8e-09 |
| Positional bias | 0.3 |
| Strand bias | 0.18 |

| Distance scores for most similar known PSSMs | |
| --- | --- |
| R02239 | 13.0 |
| S-000153 | 13.5 |
| S-000402 | 13.7 |
| MA-03491 | 14.6 |
| S-000194 | 14.6 |


Cluster 38 Motif 5

| Motif Featuresmore... | |
| --- | --- |
| Sites | 27 (8.2%) |
| Width | 11 |
| Information content | 14.6 |
| E-value | 1.2e-07 |
| Positional bias | 0.011 |
| Strand bias | 0.12 |

| Distance scores for most similar known PSSMs | |
| --- | --- |
| M00368 | 2.9 |
| M00942 | 3.4 |
| S-000041 | 3.8 |
| M00441 | 3.9 |
| M00366 | 4.1 |

  


Cluster 39 Motif 1

| Motif Featuresmore... | |
| --- | --- |
| Sites | 266 (100%) |
| Width | 12 |
| Information content | 13.1 |
| E-value | 2.8e-97 |
| Positional bias | 0.00051 |
| Strand bias | 0.079 |

| Distance scores for most similar known PSSMs | |
| --- | --- |
| S-000067 | 7.7 |
| MA-01201 | 8.8 |
| HK-00008 | 9.3 |
| EVE-0008 | 9.3 |


Cluster 39 Motif 2

| Motif Featuresmore... | |
| --- | --- |
| Sites | 59 (22.1%) |
| Width | 12 |
| Information content | 13.8 |
| E-value | 1.1e-27 |
| Positional bias | 0.095 |
| Strand bias | 0.4 |

| Distance scores for most similar known PSSMs | |
| --- | --- |
| S-000460 | 3.3 |
| MA-02051 | 7.1 |
| M00723 | 8.2 |
| R02187 | 11.6 |
| S-000427 | 11.6 |


Cluster 39 Motif 3

| Motif Featuresmore... | |
| --- | --- |
| Sites | 48 (18%) |
| Width | 7 |
| Information content | 11.6 |
| E-value | 5.3e-16 |
| Positional bias | 2.4e-05 |
| Strand bias | 0.16 |

| Distance scores for most similar known PSSMs | |
| --- | --- |
| S-000474 | 3.1 |
| S-000471 | 3.3 |
| S-000483 | 3.5 |
| MA-03811 | 7.8 |
| M00967 | 8.9 |


Cluster 39 Motif 4

| Motif Featuresmore... | |
| --- | --- |
| Sites | 79 (29.6%) |
| Width | 12 |
| Information content | 13.0 |
| E-value | 1e-09 |
| Positional bias | 5.1e-05 |
| Strand bias | 0.5 |

| Distance scores for most similar known PSSMs | |
| --- | --- |
| M01172 | 8.3 |
| M00658 | 8.5 |
| R02232 | 9.9 |
| M01126 | 9.9 |
| M00500 | 10.1 |


Cluster 39 Motif 5

| Motif Featuresmore... | |
| --- | --- |
| Sites | 48 (18%) |
| Width | 12 |
| Information content | 14.5 |
| E-value | 6.4e-12 |
| Positional bias | 0.16 |
| Strand bias | 0.0066 |

| Distance scores for most similar known PSSMs | |
| --- | --- |
| S-000362 | 9.4 |
| RJH-00017 | 10.8 |
| M00456 | 10.8 |
| S-000167 | 11.3 |
| MA-01331 | 11.9 |

  


Cluster 40 Motif 1

| Motif Featuresmore... | |
| --- | --- |
| Sites | 174 (92.5%) |
| Width | 12 |
| Information content | 13.6 |
| E-value | 3.1e-71 |
| Positional bias | 0.39 |
| Strand bias | 0.25 |

| Distance scores for most similar known PSSMs | |
| --- | --- |
| S-000067 | 8.7 |
| HK-00008 | 8.8 |
| EVE-0008 | 8.8 |
| M01021 | 9.7 |


Cluster 40 Motif 2

| Motif Featuresmore... | |
| --- | --- |
| Sites | 46 (24.4%) |
| Width | 12 |
| Information content | 13.0 |
| E-value | 8.1e-08 |
| Positional bias | 0.00012 |
| Strand bias | 0.052 |

| Distance scores for most similar known PSSMs | |
| --- | --- |
| S-000460 | 7.9 |
| MA-02051 | 8.8 |
| S-000270 | 10.4 |
| R02187 | 11.1 |
| M00723 | 11.1 |


Cluster 40 Motif 3

| Motif Featuresmore... | |
| --- | --- |
| Sites | 14 (7.4%) |
| Width | 11 |
| Information content | 12.7 |
| E-value | 3.7e+03 |
| Positional bias | 0.19 |
| Strand bias | 0.09 |

| Distance scores for most similar known PSSMs | |
| --- | --- |
| M00333 | 11.1 |
| MA-04491 | 11.5 |
| M01585 | 12.0 |
| MA-00061 | 12.0 |
| M00807 | 12.3 |


Cluster 40 Motif 4

| Motif Featuresmore... | |
| --- | --- |
| Sites | 44 (23.4%) |
| Width | 11 |
| Information content | 12.7 |
| E-value | 1.3e+03 |
| Positional bias | 0.025 |
| Strand bias | 0.33 |

| Distance scores for most similar known PSSMs | |
| --- | --- |
| S-000143 | 9.9 |
| S-000353 | 10.8 |
| MA-00061 | 11.5 |
| M01216 | 11.6 |
| M00791 | 11.7 |


Cluster 40 Motif 5

| Motif Featuresmore... | |
| --- | --- |
| Sites | 14 (7.4%) |
| Width | 11 |
| Information content | 13.9 |
| E-value | 6.6e+03 |
| Positional bias | 0.11 |
| Strand bias | 0.21 |

| Distance scores for most similar known PSSMs | |
| --- | --- |
| S-000205 | 10.0 |
| S-000325 | 11.4 |
| M01090 | 12.7 |
| S-000418 | 13.0 |
| S-000146 | 13.1 |

  


Cluster 41 Motif 1

| Motif Featuresmore... | |
| --- | --- |
| Sites | 11 (42.3%) |
| Width | 11 |
| Information content | 13.9 |
| E-value | 1.4 |
| Positional bias | 0.9 |
| Strand bias | 0.5 |

| Distance scores for most similar known PSSMs | |
| --- | --- |
| S-000460 | 9.0 |
| MA-02051 | 10.3 |
| M00723 | 10.9 |
| S-000486 | 11.1 |
| M00706 | 12.3 |


Cluster 41 Motif 2

| Motif Featuresmore... | |
| --- | --- |
| Sites | 26 (100%) |
| Width | 12 |
| Information content | 12.0 |
| E-value | 2.9e+02 |
| Positional bias | 0.49 |
| Strand bias | 0.28 |

| Distance scores for most similar known PSSMs | |
| --- | --- |
| M00438 | 9.2 |
| M00302 | 9.8 |
| S-000391 | 10.1 |
| MA-01521 | 10.2 |
| M01004 | 10.2 |


Cluster 41 Motif 3

| Motif Featuresmore... | |
| --- | --- |
| Sites | 3 (11.5%) |
| Width | 12 |
| Information content | 18.1 |
| E-value | 1.6e+04 |
| Positional bias | 0.41 |
| Strand bias | 0.5 |

| Distance scores for most similar known PSSMs | |
| --- | --- |
| S-000153 | 15.0 |
| R02239 | 15.0 |
| MA-04041 | 16.1 |
| S-000418 | 16.2 |
| S-000135 | 16.2 |


Cluster 41 Motif 4

| Motif Featuresmore... | |
| --- | --- |
| Sites | 8 (30.7%) |
| Width | 11 |
| Information content | 13.9 |
| E-value | 9e+03 |
| Positional bias | 0.24 |
| Strand bias | 0.64 |

| Distance scores for most similar known PSSMs | |
| --- | --- |
| S-000315 | 11.5 |
| MA-00861 | 11.9 |
| R04823 | 12.1 |
| M00951 | 12.2 |
| M00644 | 12.3 |


Cluster 41 Motif 5

| Motif Featuresmore... | |
| --- | --- |
| Sites | 2 (7.6%) |
| Width | 8 |
| Information content | 14.1 |
| E-value | 9.7e+04 |
| Positional bias | 0.59 |
| Strand bias | 0.75 |

| Distance scores for most similar known PSSMs | |
| --- | --- |
| M00915 | 10.3 |
| M00470 | 10.5 |
| M01160 | 10.5 |
| M01546 | 10.7 |
| M00469 | 10.7 |

  


Cluster 42 Motif 1

| Motif Featuresmore... | |
| --- | --- |
| Sites | 217 (71.8%) |
| Width | 12 |
| Information content | 13.2 |
| E-value | 5.6e-103 |
| Positional bias | 0.00027 |
| Strand bias | 0.45 |

| Distance scores for most similar known PSSMs | |
| --- | --- |
| MA-00391 | 9.9 |
| R05016 | 10.3 |
| R02187 | 10.6 |
| MA-01201 | 10.9 |
| M01126 | 11.0 |


Cluster 42 Motif 2

| Motif Featuresmore... | |
| --- | --- |
| Sites | 75 (24.8%) |
| Width | 11 |
| Information content | 12.8 |
| E-value | 1.6e-20 |
| Positional bias | 1.4e-05 |
| Strand bias | 0.018 |

| Distance scores for most similar known PSSMs | |
| --- | --- |
| S-000460 | 4.0 |
| MA-02051 | 7.1 |
| M00723 | 8.5 |
| S-000486 | 9.1 |
| MA-00391 | 10.9 |


Cluster 42 Motif 3

| Motif Featuresmore... | |
| --- | --- |
| Sites | 231 (76.4%) |
| Width | 12 |
| Information content | 12.7 |
| E-value | 6.4e-24 |
| Positional bias | 0.56 |
| Strand bias | 3.6e-05 |

| Distance scores for most similar known PSSMs | |
| --- | --- |
| S-000477 | 8.2 |
| M01216 | 9.1 |
| HK-00008 | 9.1 |
| EVE-0008 | 9.1 |


Cluster 42 Motif 4

| Motif Featuresmore... | |
| --- | --- |
| Sites | 18 (5.9%) |
| Width | 10 |
| Information content | 11.4 |
| E-value | 8.8 |
| Positional bias | 0.46 |
| Strand bias | 0.59 |

| Distance scores for most similar known PSSMs | |
| --- | --- |
| R02262 | 6.2 |
| MA-01231 | 7.6 |
| R02147 | 7.8 |
| R02148 | 8.6 |
| M01219 | 8.7 |


Cluster 42 Motif 5

| Motif Featuresmore... | |
| --- | --- |
| Sites | 25 (8.2%) |
| Width | 11 |
| Information content | 13.5 |
| E-value | 0.00056 |
| Positional bias | 0.055 |
| Strand bias | 0.054 |

| Distance scores for most similar known PSSMs | |
| --- | --- |
| S-000500 | 9.9 |
| M00506 | 11.0 |
| M00695 | 11.0 |
| M01219 | 11.5 |
| M01160 | 11.9 |

  


Cluster 43 Motif 1

| Motif Featuresmore... | |
| --- | --- |
| Sites | 208 (78.1%) |
| Width | 12 |
| Information content | 12.8 |
| E-value | 1.8e-87 |
| Positional bias | 0.00013 |
| Strand bias | 0.36 |

| Distance scores for most similar known PSSMs | |
| --- | --- |
| R02187 | 9.7 |
| MA-00391 | 10.5 |
| R05016 | 10.5 |
| M01136 | 10.8 |
| M01021 | 11.0 |


Cluster 43 Motif 2

| Motif Featuresmore... | |
| --- | --- |
| Sites | 50 (18.7%) |
| Width | 11 |
| Information content | 13.9 |
| E-value | 9.5e-15 |
| Positional bias | 0.00027 |
| Strand bias | 0.24 |

| Distance scores for most similar known PSSMs | |
| --- | --- |
| S-000471 | 3.3 |
| S-000474 | 3.9 |
| S-000483 | 6.2 |
| M00967 | 9.4 |
| M01238 | 10.1 |


Cluster 43 Motif 3

| Motif Featuresmore... | |
| --- | --- |
| Sites | 132 (49.6%) |
| Width | 11 |
| Information content | 14.1 |
| E-value | 8.8e-24 |
| Positional bias | 0.072 |
| Strand bias | 0.0034 |

| Distance scores for most similar known PSSMs | |
| --- | --- |
| S-000477 | 7.7 |
| HK-00008 | 9.1 |
| EVE-0008 | 9.1 |
| M01216 | 9.2 |


Cluster 43 Motif 4

| Motif Featuresmore... | |
| --- | --- |
| Sites | 26 (9.7%) |
| Width | 12 |
| Information content | 14.2 |
| E-value | 0.057 |
| Positional bias | 0.026 |
| Strand bias | 0.014 |

| Distance scores for most similar known PSSMs | |
| --- | --- |
| S-000225 | 10.7 |
| S-000205 | 11.7 |
| S-000402 | 12.8 |
| S-000146 | 12.9 |
| M01057 | 13.2 |


Cluster 43 Motif 5

| Motif Featuresmore... | |
| --- | --- |
| Sites | 113 (42.4%) |
| Width | 12 |
| Information content | 11.8 |
| E-value | 5.7e-06 |
| Positional bias | 0.00033 |
| Strand bias | 0.17 |

| Distance scores for most similar known PSSMs | |
| --- | --- |
| M01126 | 9.6 |
| M01295 | 10.1 |
| S-000159 | 10.6 |
| MA-01201 | 10.6 |
| M00028 | 10.6 |

  


Cluster 44 Motif 1

| Motif Featuresmore... | |
| --- | --- |
| Sites | 175 (82.1%) |
| Width | 12 |
| Information content | 12.4 |
| E-value | 8.7e-71 |
| Positional bias | 0.0066 |
| Strand bias | 0.5 |

| Distance scores for most similar known PSSMs | |
| --- | --- |
| R05016 | 9.4 |
| MA-00391 | 10.3 |
| M01126 | 10.5 |
| M01136 | 10.9 |
| M01021 | 11.3 |


Cluster 44 Motif 2

| Motif Featuresmore... | |
| --- | --- |
| Sites | 135 (63.3%) |
| Width | 11 |
| Information content | 12.7 |
| E-value | 1e-13 |
| Positional bias | 0.25 |
| Strand bias | 0.042 |

| Distance scores for most similar known PSSMs | |
| --- | --- |
| HK-00008 | 5.1 |
| EVE-0008 | 5.1 |
| S-000477 | 8.5 |
| M01216 | 8.5 |


Cluster 44 Motif 3

| Motif Featuresmore... | |
| --- | --- |
| Sites | 40 (18.7%) |
| Width | 11 |
| Information content | 13.5 |
| E-value | 7.9e-07 |
| Positional bias | 0.032 |
| Strand bias | 0.0083 |

| Distance scores for most similar known PSSMs | |
| --- | --- |
| M00438 | 9.2 |
| S-000369 | 10.2 |
| S-000337 | 10.6 |
| M00706 | 11.0 |
| S-000270 | 11.1 |


Cluster 44 Motif 4

| Motif Featuresmore... | |
| --- | --- |
| Sites | 14 (6.5%) |
| Width | 12 |
| Information content | 14.5 |
| E-value | 0.43 |
| Positional bias | 0.37 |
| Strand bias | 0.09 |

| Distance scores for most similar known PSSMs | |
| --- | --- |
| M01585 | 7.2 |
| S-000119 | 7.5 |
| MA-01042 | 7.7 |
| MA-01471 | 8.1 |
| S-000041 | 8.2 |


Cluster 44 Motif 5

| Motif Featuresmore... | |
| --- | --- |
| Sites | 45 (21.1%) |
| Width | 12 |
| Information content | 13.2 |
| E-value | 2.5e+03 |
| Positional bias | 0.2 |
| Strand bias | 0.008 |

| Distance scores for most similar known PSSMs | |
| --- | --- |
| S-000159 | 9.9 |
| M01566 | 11.1 |
| MA-03771 | 11.4 |
| MA-00811 | 11.6 |
| M01172 | 12.4 |
